# 30 Years of postdisturbance recruitment in a Neotropical forest

**DOI:** 10.1002/ece3.7634

**Published:** 2021-10-07

**Authors:** Ariane Mirabel, Eric Marcon, Bruno Hérault

**Affiliations:** ^1^ UMR EcoFoG AgroParistech CNRS Cirad INRA Université des Antilles Université de Guyane Kourou France; ^2^ CIRAD UPR Forêts et Sociétés Yamoussoukro Côte d'Ivoire; ^3^ Forêts et Sociétés Univ Montpellier CIRAD Montpellier France; ^4^ Institut National Polytechnique Félix Houphouët‐Boigny INP‐HB Yamoussoukro Côte d'Ivoire

**Keywords:** community composition, community diversity, disturbance response, functional trait, Neotropical forests, postdisturbance recovery, recruitment, successional pathway, taxonomy, tree community

## Abstract

**Questions:**

Long‐term community response to disturbance can follow manifold successional pathways depending on the interplay between various recruitment processes. Analyzing the succession of recruited communities provides a long‐term perspective on forest response to disturbance. Specifically, postdisturbance recruitment trajectories assess (a) the successive phases of postdisturbance response and the role of deterministic recruitment processes, and (b) the return to predisturbance state of recruits taxonomic/functional diversity/composition.

**Location:**

Amazonian rainforest, Paracou station, French Guiana.

**Methods:**

We analyzed trajectories of recruited tree communities, from twelve forest plots of 6.25 ha each, during 30 years following a disturbance gradient that ranged from 10% to 60% of aboveground biomass removed. We measured recruited community taxonomic composition turnover, compared to whole predisturbance community, and assessed their functional composition by measuring the community weighted means for seven leaf, stem, and life‐history functional traits. We also measured recruited community taxonomic richness, taxonomic evenness, and functional diversity and compared them to the diversity values from a random recruitment process.

**Results:**

While control plots trajectories resembled random recruitment trajectories, postdisturbance trajectories diverged significantly. This divergence corresponded to an enhanced recruitment of light‐demanding species that became dominant above a disturbance intensity threshold. After breakpoints in time, though, recruitment trajectories returned to diversity values and composition similar to those of predisturbance and control plots community.

**Conclusions:**

Following disturbance, recruitment processes specific to undisturbed community were first replaced by the emergence of more restricted, deterministic recruitment processes favoring species with efficient light use and acquisition. Then, a second phase corresponded to a decades‐long recovery of recruits predisturbance taxonomic and functional diversity and composition that remained unachieved after 30 years.

## Graphical abstract

We examined recruited trees taxonomic/functional diversity/composition trajectories in a Neotropical forest to identify the processes underlying community response to a disturbance gradient. We revealed a two‐phase successional pathway defined by (a) the emergence of deterministic recruitment processes enhancing the recruitment of light‐demanding species and (b) a decades‐long recovery towards the pre‐disturbance composition and diversity values.

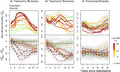

## 1 INTRODUCTION

Determining the response of tropical forests to disturbance is key for predicting their fate in the context of global climate change. In recent decades, tropical forests have experienced a wide range of disturbances, from radical land‐use changes for agriculture or mining (Dezécache, Faure, et al., 2017; Dezécache, Salles, et al., 2017) to more insidious changes following climate change or human activities like selective logging (Aubry‐Kientz et al., 2015; Baraloto et al., 2012). In that respect, a vast literature has successfully modeled community response to disturbance in terms of tree growth, tree height, and fluxes of carbon, water, and nutrients (Gourlet‐Fleury & Houllier, 2000; Piponiot et al., 2016; Putz et al., 2012; Rutishauser et al., 2016). Tree community diversity and composition, however, proved more complex to predict as forest succession is driven both by deterministic, predictable, and by random, varying processes (Brokaw & Busing, 2000; Norden et al., 2015). Deterministic processes would correspond to recruitment processes depending on the species resources use strategy and competitive abilities. Following disturbance, species benefiting the most from higher resources availability and reduced competition would be favored in the first place (Adler et al., 2007; Chesson, 2000; Rees et al., 2001), before stand maturation progressively allows late‐successional species to recruit and restore predisturbance composition and diversity (Denslow & Guzman, 2000). In the very diverse tropical forests, however, while several studies revealed predictable and homogeneous successional phases restoring predisturbance community characteristics (Letcher et al., 2015), others revealed diverging postdisturbance trajectories and different equilibrium states (Longworth et al., 2014). Specifically, the Neotropical forests studied here revealed complex trajectories at the scale of the whole community, that is, considering both recruited and predisturbance surviving trees, with a divergence between taxonomic and functional trajectories and a challenging estimation of the time to recover initial state (Mirabel et al., 2020). Following on these conclusions, a focus on recruitment trajectories would better assess the differences among successional pathways and the corresponding recruitment processes. Clarifying recruitment trajectories would also help to give an idea of what would be the diversity and composition of future communities (Clark et al., 1998; Grubb, 1977; Hurtt & Pacala, 1995). We aimed here to focus on recruitment processes driving a Neotropical forest response to disturbance: We assessed the successional phases of postdisturbance recruitment (Chazdon, 2008; Norden et al., 2015) and determined whether they allow the recovery of community predisturbance state.

Two major facets of communities’ description are their taxonomic characteristics, corresponding to species composition and diversity, and their functional characteristics, accounting for species ecology and functioning (Kunstler et al., 2016; MacArthur & Levins, 1967; Violle et al., 2007). Deterministic processes correspond to a recruitment depending on species competitive ability determined by their functional characteristics (Perronne et al., 2017; Rees et al., 2001). In wet tropical forests, where light is hypothesized to be the main limiting resource, deterministic processes might be revealed by community functional trajectories considering a set of key functional traits assessing species ecology and resources acquisition strategy, such as leaf, wood, and life‐history functional traits (Chave et al., 2009; Hérault et al., 2011; Wright et al., 2004). Such studies have revealed a successional sequence in functional composition from fast‐growing species with “acquisitive” resource use, capable of significant and rapid acquisition of light, to slow‐growing, long‐lived species with “conservative” resource use (Bongers et al., 2009; Denslow, 1980; Molino & Sabatier, 2001). Then, while deterministic recruitment would result in taxonomic and functional trajectories restricted to certain species or functional strategies, nondeterministic recruitment would correspond to a recruitment independent of species functional characteristics. A combination of taxonomic and functional approaches can thus reveal both deterministic and nondeterministic recruitment processes driving community response to disturbance (Cequinel et al., 2018; Chalmandrier et al., 2015; Fukami et al., 2005).

Here, we followed recruitment trajectories in a 75‐ha area, cumulated over experimental plots, of Neotropical forest subject to a gradient of disturbance intensities. The gradient corresponded to a removal by logging and girdling of 10%–60% of aboveground standing biomass. We first investigated community composition, richness, and evenness of recruited trees over 30 years, and their taxonomic composition turnover compared to the predisturbance community. We considered both taxonomic aspects, that is, tree species diversity and composition, and functional aspects, accounting for seven major leaf, stem, and life‐history traits. Breakpoints analysis allowed us to specify the different phases of the observed trajectories. We compared the recruitment trajectories to a nondeterministic recruitment, obtained from null models corresponding to a random sampling of recruits and a randomization of species functional traits. Specifically, we (a) determined postdisturbance successional phases and analyzed the role of deterministic processes along time and (b) examined the return of recruited community to predisturbance taxonomic and functional characteristics.

## 2 MATERIAL AND METHODS

### 2.1 Study site

The Paracou station is located in a lowland tropical rainforest in French Guiana (5°18′N and 52°53′W). The climate is tropical wet with mean annual precipitation averaging 2,980 mm/year (30‐year period) and a 3‐month dry season (<100 mm/month) from mid‐August to mid‐November and a one‐month dry season in March (Wagner et al., 2011). The mean annual temperature is 26°C. Elevation ranges from 5 to 50 m. Across all the study plots, the topography mainly corresponds to hilltops or hillsides, while bottomlands account for <1% of the area. The plots contain shallow ferralitic acrisols over a layer of transformed saprolite with low permeability and lateral drainage. Soil conditions are homogeneous, with the exception of the highest hilltops where the thick surface soil layer allows free vertical drainage (Gourlet‐Fleury et al., 2004).

The experiment comprises a network of twelve 6.25‐ha plots (Table 1) with three replicates of three disturbance treatments applied in 1987 according to a randomized plot design (Hérault & Piponiot, 2018), and three control plots. The treatments included successively, along the intensity gradient, the logging of a set of 58 commercially exploited species, the thinning by poison‐girdling of noncommercially exploited species, with thinned trees left standing in the plots, and eventually the additional logging of noncommercially exploited species (Gourlet‐Fleury et al., 2004). Disturbance intensity was measured considering the silvicultural treatments and the resulting damage through the percentage of aboveground standing biomass of trees above 10 cm DBH (%AGB) lost between the first inventory in 1984 and that conducted five years after disturbance, when we considered that tree mortality induced by disturbance had ended (Piponiot et al., 2016). Above ground biomass was estimated using the BIOMASS R package (Réjou‐Méchain et al., 2017, 2018), without accounting for lianas.

**TABLE 1:** Intervention table, summary of the disturbance intensity for the four plot treatments in Paracou

Treatment	Timber	Thinning	Fuelwood	%AGB lost
Control	–	–	–	0
T1	50 cm ≤ DBH, Commercial species, ≈10 trees.ha^−1^	–	–	[12–33]
T2	50 cm ≤ DBH, Commercial species, ≈10 trees.ha^−1^	40 cm ≤ DBH, nonvaluable species, ≈30 trees.ha^−1^	–	[33–56]
T3	50 cm ≤ DBH, Commercial species, ≈10 trees.ha^−1^	50 cm ≤ DBH, nonvaluable species, ≈15 trees.ha^−1^	40 cm ≤ DBH ≤ 50 cm, nonvaluable species, ≈15 trees.ha^−1^	[35–56]

### 2.2 Inventory protocol and data collection

Dominant families in the study site were Fabaceae, Chrysobalanaceae, Lecythidaceae, and Sapotaceae. All trees above 10 cm DBH were mapped and measured annually since 1984. The trees are first identified with a vernacular name attributed by the technical staff and subsequently with a scientific name assigned by botanists during regular botanical campaigns. Botanical campaigns were carried out at five‐ to six‐year intervals starting in 2003, but identification accuracy, that is, at species, gender, or family level, varied between campaigns. The variability of protocols over time raised methodological issues as the vernacular names usually corresponded to different botanical species. This variability resulted in significant taxonomic uncertainties that had to be extended to composition and diversity metrics. Uncertainty propagation was performed using a Bayesian framework reconstructing complete inventories at species level from real incomplete ones through the link between vernacular and botanical names. Vernacular names were replaced through multinomial trials based on the association probability $\left[\alpha_{1},\alpha_{2},\ldots ,\alpha_{N}\right]$ observed across all inventories between each vernacular name *v* and the species $\left[s_{1},s_{2},\ldots ,s_{N}\right]$:

$$\begin{array}{c} M_{v}\left(\left[s_{1},s_{2},\ldots ,s_{N}\right],\left[\alpha_{1},\alpha_{2},\ldots ,\alpha_{N}\right]\right) \end{array}$$

See Appendix S1 and Aubry‐Kientz et al. (2013), for the detailed methodology.

We considered six functional traits representing leaf economics: leaf thickness, toughness, total chlorophyll content, and specific leaf area; and stem economics: wood‐specific gravity and bark thickness. These traits were obtained from the BRIDGE project (http://www.ecofog.gf/Bridge/), which reliably measured species functional traits and community functional diversity (Paine et al., 2015), as detailed below. Trait values were measured from a selection of individuals located in nine permanent plots in French Guiana, including two in Paracou, and comprised 294 species belonging to 157 genera. Between 1 and 159 individuals were sampled per species, and for each individual, three new but fully expanded leaves were collected. Collected leaves were used for measurement of the leaf thickness, measured with a digital micrometer on three points per leaf; the leaf toughness, measured with a digital penetrometer on three points per leaf; the leaf chlorophyll content, measured with a SPAD meter on three points per leaf and converted with calibrations for French Guianan tropical trees (Coste et al., 2010); and the leaf specific area, computed as the ratio between leaf area measured from digital scan and dry mass measured following drying to constant mass at 50°C. Mean foliar trait values of sampled leaves were used as tree‐level trait value. Trunk samples were collected at 1 m high to measure bark thickness and wood‐specific gravity. The later was measured, after removal of the bark, and was computed as the ratio between wood dry mass measured following 72 hr drying at 103°C and fresh volume measured by the water displacement method. For the trait value at species level, we used the median value of all the trees in the database. The table of median trait values per species is available in Appendix S4.

In the dataset, 49% of the individuals and 13% of the species sampled during the traits measurement campaign had missing trait values, that is, some traits are informed while others are not. The missing values were filled in using multivariate imputation by chained equations (van Buuren & Groothuis‐Oudshoorn, 2011). To account for the phylogenetic signal in the imputation process, imputations were based on samples from the same genus or from the same family. Besides, 40% of the species observed in the plot were not recorded in the trait dataset, that is, they were not sampled during the traits measurement campaign, which represents 15% of the observed individuals. For these species, we did not have any trait information in hand. Whenever a species was not in the trait dataset, it was attributed a set of trait values randomly sampled among species at the next higher taxonomic level (same genus or family). In addition, maximum specific height was retrieved from the Mariwenn database (https://www.ecofog.gf/mariwenn/). This database compiles information from the floristic literature on French Guiana (Ollivier et al., 2007) and comprises 362 species belonging to 188 genera.

All composition and diversity metrics were obtained after 100 iterations of the uncertainty propagation framework.

### 2.3 Recruitment trajectories

To explore recruitment trajectories in time, trees that survived the disturbance were discarded from the following analyses. Our observation of recruitment diversity trajectories and turnover started from the fifth year after disturbance, when we considered that tree mortality induced by disturbance had ended (Piponiot et al., 2016). Trajectories then considered the trees recruited at 2‐year intervals (i.e., for time *T*, the trees that were under 10 cm DBH 2 years before but above 10 cm DBH at *T*). For control plots, the starting point of recruitment trajectories was the same year as for disturbed plots, that is, five years after disturbance was applied.

Taxonomic diversity (*H* ^q^_obs_) trajectories were examined through species richness and the Hill number transformation of the Simpson index assessing communities evenness. The later will be designated as “taxonomic evenness” (Chao & Jost, 2015; Marcon & Hérault, 2015). The two diversity measures derived from the set of generalized entropy measures, also called Tsallis entropy, and respectively correspond to the zero and two order of diversity. The order of diversity emphasizes species importance according to their stem density. Community richness and evenness account for the structure of dominance in the community and their use can measure changes in community abundance distribution. The two diversity metrics are complementary for the detection of changes in community structure (Magurran, 2004) and will directly be referred to as richness and evenness thereafter.

Functional diversity trajectories were assessed using the Rao index of quadratic entropy, which combines species abundance distribution and average pairwise dissimilarity based on all measured functional traits:

$$H_{\Delta }\left(P\right)=\sum_{s^{\prime }}\sum_{s^{\prime \prime }}p_{s^{\prime }}p_{s^{\prime \prime }}d_{s^{\prime }s^{\prime \prime }}$$

With $p_{s^{\prime }}$ $p_{s^{\prime \prime }}$ the frequency of species $s^{\prime }$ and $s^{\prime \prime }$ in the community *P*, and Δ the functional dissimilarity matrix which elements $d_{s^{\prime }s^{\prime \prime }}$ are the Euclidean distances between species measured from species trait values.

The observed recruitment trajectories were compared to random trajectories obtained from the taxonomic and functional null models detailed below. A taxonomic null model was built independently for each plot and each year. Taxonomic null trajectories were simulated by resampling the recruits in the community of the year and plot considered. The number of trees sampled was the same as the observed number of recruits. This model aimed to simulate an assemblage of species similar to this of the whole community in terms of community abundance distribution. Functional null trajectories were simulated by randomizing the (species × trait) matrix, so combinations of trait values were randomly reassigned between species. The functional null models maintained the community abundance distribution while randomizing the relative abundance of trait values (Mason et al., 2013). Trajectories of null diversity (hereafter *H* ^q^_null_) were similarly obtained after 100 iterations of the random sampling models. For each iteration of the null model, we computed the difference between null and observed trajectories. The 100 iterations made it possible to calculate a 95% confidence interval of the difference between null and observed trajectories.

We tested whether disturbance intensity, in terms of AGB lost, was correlated with diversity and composition using Spearman's test. Specifically, changes in taxonomic richness, taxonomic evenness, and functional richness were assessed as the maximum difference over the 30 years between observed and predisturbance values and between observed and null models’ values. Changes in taxonomic turnover were assessed as the maximum taxonomic turnover value over the 30 years.

Functional composition trajectories were assessed through community weighted means (CWM) of functional traits. CWM are average trait values in the community weighted by the species relative abundance of the number of stems (Díaz et al., 2007; Figure 2). A table of trait correlations is provided in Appendix S1 for a better overview of the trade‐offs among traits. In the graphs (Figure 2), CWM trajectories are represented by the fitted lines of generalized additive models (GAM) adjusted at plot level. GAM represents the nonlinear response of functional trait value as the addition of basic smoothing functions. The number of smoothing functions and the smoothing parameters were selected using the restricted maximum‐likelihood method in the mgcv R package (Wood, 2011).

Taxonomic composition trajectories compared the taxonomic composition of recruited communities to the whole predisturbance community, which allowed removing the strong taxonomic signature of the plots. Taxonomic composition trajectories corresponded to the taxonomic similarity between recruited trees and the whole predisturbance community, including all trees surviving from disturbance. The taxonomic similarity was measured with the relativized abundance replacement measuring species turnover between communities (Podani et al., 2013):

$$T_{\mathrm{ab}}=\frac{\sum_{i=1}^{n}\left|x_{i}^{a}‐x_{i}^{b}\right|‐\left|\sum_{i=1}^{n}x_{i}^{a}‐\sum_{i=1}^{n}x_{i}^{b}\right|}{\sum_{i=1}^{n}\max(x_{i}^{a};x_{i}^{b})}$$

With *T_ab_* the turnover between communities *a* and *b*, *n* the total number of species in the two communities and *x^a^* ^/^ *^b^_i_* the abundance of species *i* in community *a* or *b*.

Postdisturbance diversity, composition, and turnover trajectories were analyzed to detect their shifts via breakpoints analysis derived from methods developed in Bai and Perron (2003). Observed trajectories were partitioned into two or three intervals, each fit by linear regression models. Intervals were defined by one or two different points in time, representing potential breakpoints of the trajectories. For each trajectory, the number and time of breakpoints and the corresponding linear models that minimized the mean square error for the whole trajectory were retained.

## 3 RESULTS

The average number of recruited trees per plot and per year throughout the survey increased with increasing disturbance intensity. Over the 30‐year period, a total of 602 species were recruited across the 12 plots. Among these species, 26% only occurred in one plot and 7% occurred in all plots.

**FIGURE 1:**
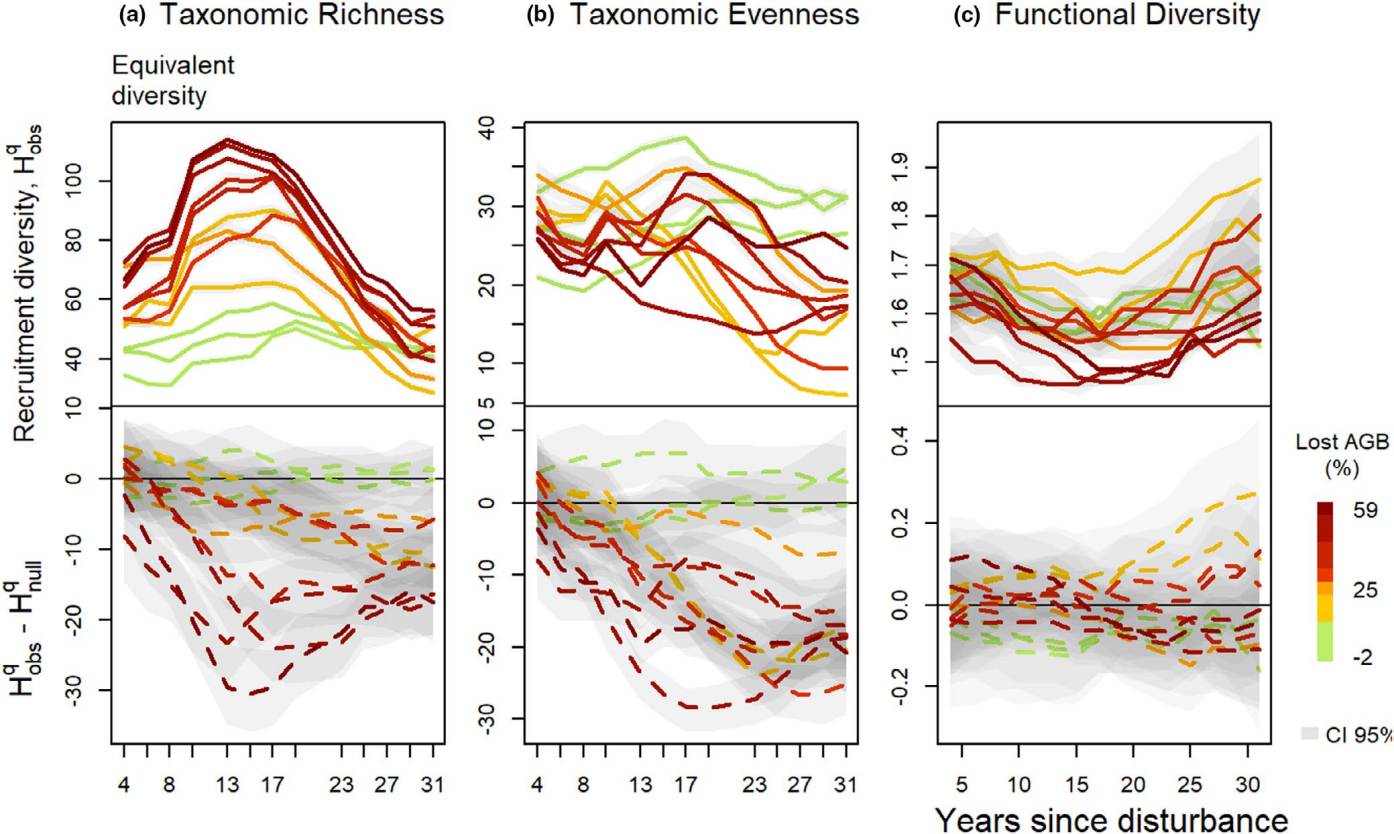
Trajectories over 30 years of taxonomic richness (a), taxonomic evenness (b), and functional diversity (c) of the communities recruited at 2‐year intervals. Colored lines stand for the disturbance intensity in percentage of AGB lost, including control plots in green. Upper panels show observed at plot level, *H* ^q^_obs_, and the lower panels show the difference between observed values and null values models *H* ^q^_obs_–*H* ^q^_null_. Shaded areas are the 95% confidence intervals, CI 95%, measured from the 100 iterations of the null models, when not visible on the figure, intervals are too small

The propagation framework for botanical uncertainties returned an estimation of community diversity with a 95% confidence interval that remained below 10% of the estimated value. Taxonomic diversity estimations showed significantly distinct trajectories among plots with no overlapping confidence intervals (Figure 1a,b), while functional trajectories were not as distinctly separated (Figure 1c),

### 3.1 Taxonomic richness and evenness

In undisturbed communities, the taxonomic richness and evenness of the recruited trees remained stable over the 30‐year period, with values equivalent to those of the taxonomic null model (Figure 1a,b). In disturbed communities, the taxonomic richness followed hump‐shaped trajectories first increasing and reaching maximum after around 15 years. This maximum was positively correlated with disturbance intensity ($\rho_{\text{Spearman}}^{\text{Richness}}$ = 0.87, *p*‐value = 3.1 × 10^−4^). After the maximum was reached, taxonomic richness decreased and recovered predisturbance values after 30 years. The comparison with the null models showed an observed taxonomic richness in disturbed plots significantly lower than what would be expected from the observed community. The confidence interval of the difference between observed and null models remained well under zero in all disturbed plots, and the maximum difference was positively correlated with disturbance intensity ($\rho_{\text{Spearman}}^{\text{Richness}}=0.83$, *p*‐value = 1.7 × 10^−3^). The breakpoints analysis of taxonomic richness trajectories revealed a single breakpoint occurring between 15 and 20 years following disturbance (Appendix S2).

In all disturbed communities, the taxonomic evenness decreased over the 30 years whenever the disturbance intensity, but the maximum decrease was not correlated with disturbance intensity ($\rho_{\text{Spearman}}^{\text{Evenness}}$ = 0.03, *p*‐value = 9.2 × 10^−1^). The comparison with the null model showed an observed taxonomic evenness significantly lower than what would be expected from the observed community. The difference between the observed and null model evenness followed a hump‐shaped trajectory with a maximum difference reached between 20 and 25 years after disturbance, and the maximum difference was positively correlated with disturbance intensity ($\rho_{\text{Spearman}}^{\text{Evenness}}=0.45$, *p*‐value = 1.5 × 10^−1^). The breakpoint analysis of evenness trajectories revealed a single breakpoint occurring between 10 and 23 years following disturbance (Appendix S2).

### 3.2 Functional diversity

The functional diversity in the undisturbed plots changed little and remained equivalent to that of the functional null model over the 30‐year period. In the plots with the least disturbance, below 30% of lost AGB, functional diversity remained stable or increased slightly and, for two plots, was higher than that of the null model. In disturbed plots with higher disturbance intensity, functional diversity decreased until 15 years after disturbance and then started to recover initial values (Figure 1c). The maximum change in community functional diversity over the 30 years was positively correlated with the disturbance intensity (*ρ* ^Functional diversity^ = 0.24, *p*‐value = 4.4 × 10^−1^). The observed functional diversity remained lower than that of the null model over the 30‐year period (Figure 1c). The maximum difference between observed and null trajectories was positively but not significantly correlated with the disturbance intensity (*ρ* ^Functional diversity^ = 0.06, *p*‐value = .8). The breakpoints point analysis of functional diversity revealed a single breakpoint occurring from 10 to 23 years following disturbance (Appendix S2).

### 3.3 Functional composition

**FIGURE 2:**
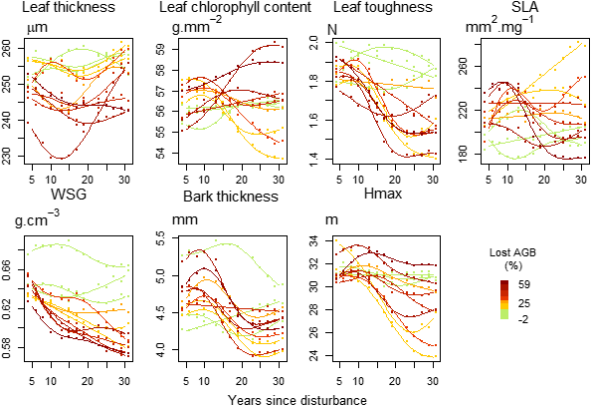
The trajectories of community weighted means (CWM) over the 30‐year period of the seven functional traits measured in the populations recruited by 2‐years intervals. Lines represent the observed trajectories fit from GAM models at plot level and the colors stand for the disturbance intensity in percentage of AGB lost, including control plots in green. From top left to bottom right, the graphs correspond to leaf thickness, leaf chlorophyll content, leaf toughness, specific leaf area (SLA), wood specific gravity (WSG), bark thickness and maximum height at the adult stage (*H*_max_)

Functional traits CWM in undisturbed plots remained stable over the 30‐year period whereas in all the disturbed plots traits CWM trajectories were hump‐shaped, with the exception of leaf chlorophyll content (Figure 2). The trajectories of SLA and bark thickness initially increased before decreasing toward initial values. Conversely, trajectories of leaf thickness, leaf toughness, wood‐specific gravity, and maximum height first decreased and then started returning to their initial values but their recovery was still not complete at the end of the 30‐year period (Figure 2).

### 3.4 Recruitment turnover

**FIGURE 3:**
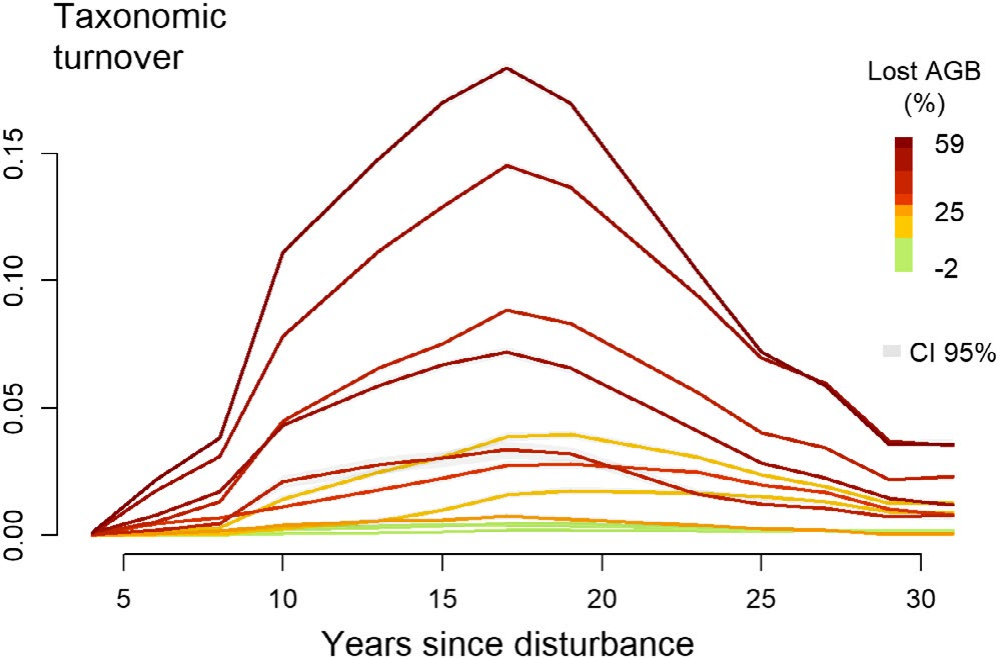
Trajectories over the 30 years of the relativized abundance‐based turnover between recruited populations and the whole pre‐disturbance communities. Recruited populations were analyzed at 2‐year intervals at plot level and compared to the plot inventory carried out before the disturbance. The colored lines stand for the disturbance intensity in precentage of AGB lost, including control plots in green. Shaded areas are the 95% confidence intervals, CI 95%, measured from the 100 iterations of the null models, when not visible on the figure, intervals are too small

The turnover of recruited species in control plots compared to the initial community remained low over the 30‐year period (Figure 3). In disturbed plots, the turnover of the recruited species followed a marked hump‐shaped trajectory, with a maximum reached about 15 years after the disturbance. The maximum turnover was positively correlated with disturbance intensity ($\rho_{\text{Spearman}}^{\text{Turnover}}$ = 0.93, *p*‐value = 1.3 × 10^−5^). The breakpoints analysis of taxonomic turnover revealed a single breakpoint occurring from 17 to 23 years following disturbance (Appendix S2). Thirty years after the disturbance, the turnover had returned to low values.

The dominant recruited species were *Lecythis persistens*, *Licania alba*, *Oenocarpus bataua*, *Oxandra asbeckii*, and *Eperua grandiflora*. The dominant species recruited in disturbed plots were *Miconia tschudyoides*, *Inga* sp., *Oenocarpus bataua*, *Licania alba*, and *Xylopia* sp.. We besides analyzed the dominant species of recruited communities in disturbed plots, considering the years before or after the breakpoints detected for each plot trajectory with the breakpoint analysis. During the years before the breakpoints, the dominant species recruited in disturbed plots were *Vismia* spp., *Cecropia obtusa*, *Oxandra asbeckii*, *Cecropia sciadophylla*, and *Lecythis persistens*. From the years following the breakpoints, the dominant species were *Miconia tschudyoides*, *Miconia acuminata*, *Oenocarpus bataua*, *Oxandra asbeckii,* and *Xylopia nitida*. The complete list of species inventoried is presented in appendix (Appendix S3).

## 4 DISCUSSION

Our study showed that recruitment trajectories followed a two‐phased successional pathway. First, trajectories were defined by the emergence, a decade after disturbance, of deterministic competition for light. Then, nondeterministic, broader recruitment processes recovered progressively (Clements, 1916; Denslow & Guzman, 2000; Meiners et al., 2015). The first phase, which length and extent depended on the disturbance intensity, corresponded to the dominance of pioneers and light‐demanding species in recruited communities. The second phase was a progressive return to the recruitment processes specific to undisturbed communities, corresponding to a dominance of shade‐tolerant, late‐successional species.

The propagation framework of botanical uncertainties proved accurate enough to discriminate and analyze postdisturbance trajectories of recruited communities in the long‐term trends.

### 4.1 The emergence of deterministic successional pathway

A first phase was defined by an increase of the taxonomic turnover between recruited trees and initial communities (Figure 3) and by the divergence between observed and null trajectories (Figure 1). Just after disturbance, the functional composition shifted from a broad panel of species functional strategies to more “resource‐acquisitive” species (Figure 2). Recruits specifically displayed higher light acquisition efficiency, that is, high SLA and low maximum height, leaf toughness, and wood‐specific gravity (Chave et al., 2009; Hérault et al., 2011; Wright et al., 2004). This shift toward “light‐acquisitive” functional strategies was coherent with the assumed role of light availability in the successional pathways followed by postdisturbance recruitment processes, as already demonstrated in temperate and tropical forests (Both et al., 2019; Carreño‐Rocabado et al., 2012; Kunstler et al., 2016; Peña‐Claros et al., 2008). During the first decades following disturbance, recruits comprised mainly species belonging to *Cecropia* or *Vismia* genera, recognized as pioneers in former studies, or species belonging to *Miconia*, *Oxandra*, or *Xylopia* genera, recognized as nonpioneer light‐demanding species (Fortunel et al., 2014; Molino & Sabatier, 2001). Postdisturbance trajectories hence revealed, during a first phase, the emergence of deterministic recruitment processes based on species light acquisition strategy which favored only a restricted pool of pioneers and light‐demanding species (Figures 1 and 2). These postdisturbance recruitment processes resulted in a decrease of the taxonomic richness and evenness of the recruited communities. Changes in community diversity and composition were all the more significant that disturbance had been intense, suggesting an increasing importance of postdisturbance deterministic processes (Bongers et al., 2009).

### 4.2 Return toward predisturbance recruitment processes

Following the first phase, lasting between 15 and 20 years depending on the disturbance intensity, recruitment trajectories showed a return toward predisturbance values of functional diversity and taxonomic richness (Fortunel et al., 2014; Fukami et al., 2005). Taxonomic evenness and functional composition, however, remained altered (Figures 1 and 2). The second phase corresponded to the recruitment of both species with “acquisitive” strategies, resulting in an increase of leaf thickness and leaf toughness CWM values, and of species with “conservative” strategies, resulting in an increase of maximum height and bark thickness CWM values (Figure 2). The changes in functional trajectories matched the changes in taxonomic composition. Although pioneers and light‐demanding species were still dominant in recruited communities, as species belonging to *Inga* or *Miconia* genera, late‐successional species as *Licania alba* progressively established (Fortunel et al., 2014). The recruitment of late successionals could reflect the progressive closing of the forest canopy and the increase in competition for light and space (Denslow & Guzman, 2000; Peet, 1992). During the second successional phase, lowly restrictive recruitment processes, specific to undisturbed forests, progressively offset the first postdisturbance deterministic processes (Chave, 2004; Lawton & Putz, 1988).

Does it mean that such a return toward predisturbance state would result in values of diversity and composition identical to the predisturbance community? Our results do not allow answering that, but they are coherent with the idea of significant dispersal limitations among tropical tree species, without which other species might have colonized the community and changed its composition and diversity (Svenning & Wright, 2005). Anyway, the general trend in return toward predisturbance state allows to take more perspectives on previous results obtained in the Paracou experiment, conducted 10 years (Molino & Sabatier, 2001) and 20 years (Baraloto et al., 2012) after disturbance. Both studies showed the consistent changes in community taxonomic and functional composition but the second study, conducted later, showed lower contrasts among disturbed plots. This was interpreted as early signs of a return toward predisturbance states, as species detected in the first study were short‐lived pioneers that did not persist until the second study. Our results confirm that these early signs corresponded well to a profound change in community trajectories. Although community trajectories returned toward predisturbance states, both taxonomic and functional characteristics remained different from predisturbance community and from control plot values 30 years after the disturbance. The higher the disturbance intensity, the more persistent the dominance of light‐demanding species. Such a long‐term impact raises questions for the management of tropical forests as most commercially valuable species are late‐successional species. Their exploitation would consequently require cutting cycles longer than 30 years, as it is currently applied in most tropics, as after this duration no plot had returned to predisturbance value (Putz et al., 2012). Furthermore, persistent changes in community composition likely alter community functioning (Díaz et al., 2005), and increase the risk of losing keystone species, with unexpected ecological consequences (Chazdon, 2003; Jones et al., 1994). Infrequent species might indeed have unique functional characteristics in the ecosystem, apart from the ones considered here, or be a key resource for some of the fauna (Schleuning et al., 2016).

## 5 CONCLUSION

Postdisturbance recruitment trajectories revealed a two‐phase deterministic successional pathway driven by the emergence of trait‐based deterministic processes favoring light‐acquisitive species. The first phase corresponded to the recruitment of a restricted pool of pioneers and light‐demanding species benefiting from the emergence of competitive exclusion for light. Above a disturbance intensity threshold, disturbed communities saw the dominance of short‐lived, fast‐growing pioneers that drastically changed community composition, diversity, and likely functioning of recruits. The second phase corresponded to the progressive recovery of broader recruitment processes specific to undisturbed communities and to a shift toward predisturbance taxonomic and functional diversity and composition. However, the recovery toward predisturbance diversity and composition values took longer than 30 years after the original disturbance. Concerning forest management, our results support cutting cycles longer than 30 years and demonstrate long‐term impacts, underlining the need to evaluate forest management sustainability.

## CONFLICT OF INTEREST

None declared.

## AUTHOR CONTRIBUTIONS

**Ariane Mirabel:** Conceptualization (equal); Formal analysis (lead); Methodology (lead); Writing‐original draft (lead). **Eric Marcon:** Conceptualization (equal); Formal analysis (supporting); Methodology (supporting); Writing‐review & editing (equal). **Bruno Hérault:** Conceptualization (equal); Formal analysis (supporting); Methodology (supporting); Writing‐review & editing (equal).

## Supporting information

Appendix S1

Appendix S2

Appendix S3

Appendix S4

## Data Availability

This article is based upon the Paracou station dataset, which is part of the Guyafor permanent plots network in French Guiana (Cirad‐CNRS‐ONF). The dataset is available from the scientific director (https://paracou.cirad.fr) upon request.

## References

[ece37634-bib-0001] Adler, P. B. , HilleRislambers, J. , & Levine, J. M. (2007). A niche for neutrality. Ecology Letters, 10(2), 95–104. 10.1111/j.1461-0248.2006.00996.x 17257097

[ece37634-bib-0002] Aubry‐Kientz, M. , Hérault, B. , Ayotte‐Trépanier, C. , Baraloto, C. , & Rossi, V. (2013). Toward trait‐based mortality models for tropical forests. PLoS One, 8(5), e63678. 10.1371/journal.pone.0063678 23675500 PMC3652824

[ece37634-bib-0003] Aubry‐Kientz, M. , Rossi, V. , Wagner, F. , & Hérault, B. (2015). Identifying climatic drivers of tropical forest dynamics. Biogeosciences, 12(19), 5583–5596. 10.5194/bg-12-5583-2015

[ece37634-bib-0004] Bai, J. , & Perron, P. (2003). Computation and analysis of multiple structural change models. Journal of Applied Econometrics, 18(1), 1–22. 10.1002/jae.659

[ece37634-bib-0005] Baraloto, C. , Hérault, B. , Paine, C. E. T. , Massot, H. , Blanc, L. , Bonal, D. , Molino, J.‐F. , Nicolini, E. A. , & Sabatier, D. (2012). Contrasting taxonomic and functional responses of a tropical tree community to selective logging. Journal of Applied Ecology, 49(4), 861–870. 10.1111/j.1365-2664.2012.02164.x

[ece37634-bib-0006] Bongers, F. , Poorter, L. , Hawthorne, W. D. , & Sheil, D. (2009). The intermediate disturbance hypothesis applies to tropical forests, but disturbance contributes little to tree diversity. Ecology Letters, 12(8), 798–805. 10.1111/j.1461-0248.2009.01329.x 19473218

[ece37634-bib-0007] Both, S. , Riutta, T. , Paine, C. E. T. , Elias, D. M. O. , Cruz, R. S. , Jain, A. , Johnson, D. , Kritzler, U. H. , Kuntz, M. , Majalap‐Lee, N. , Mielke, N. , Montoya Pillco, M. X. , Ostle, N. J. , Arn Teh, Y. , Malhi, Y. , & Burslem, D. F. R. P. (2019). Logging and soil nutrients independently explain plant trait expression in tropical forests. New Phytologist, 221(4), 1853–1865. 10.1111/nph.15444 30238458

[ece37634-bib-0008] Brokaw, N. , & Busing, R. T. (2000). Niche versus chance and tree diversity in forest gaps. Trends in Ecology & Evolution, 15(5), 183–188. 10.1016/S0169-5347(00)01822-X 10782131

[ece37634-bib-0009] Carreño‐Rocabado, G. , Peña‐Claros, M. , Bongers, F. , Alarcón, A. , Licona, J.‐C. , & Poorter, L. (2012). Effects of disturbance intensity on species and functional diversity in a tropical forest. Journal of Ecology, 100(6), 1453–1463. 10.1111/j.1365-2745.2012.02015.x

[ece37634-bib-0010] Cequinel, A. , Capellesso, E. S. , Marcilio‐Silva, V. , Cardoso, F. C. G. , & Marques, M. C. M. (2018). Determinism in tree turnover during the succession of a tropical forest. Perspectives in Plant Ecology, Evolution and Systematics, 34, 120–128. 10.1016/j.ppees.2018.08.007

[ece37634-bib-0011] Chalmandrier, L. , Münkemüller, T. , Lavergne, S. , & Thuiller, W. (2015). Effects of species’ similarity and dominance on the functional and phylogenetic structure of a plant meta‐community. Ecology, 96(1), 143–153. 10.1890/13-2153.1 26236899 PMC4539579

[ece37634-bib-0012] Chao, A. , & Jost, L. (2015). Estimating diversity and entropy profiles via discovery rates of new species. Methods in Ecology and Evolution, 6(8), 873–882. 10.1111/2041-210X.12349

[ece37634-bib-0013] Chave, J. (2004). Neutral theory and community ecology. Ecology Letters, 7(3), 241–253. 10.1111/j.1461-0248.2003.00566.x

[ece37634-bib-0014] Chave, J. , Coomes, D. , Jansen, S. , Lewis, S. L. , Swenson, N. G. , & Zanne, A. E. (2009). Towards a worldwide wood economics spectrum. Ecology Letters, 12, 351–366. 10.1111/j.1461-0248.2009.01285.x 19243406

[ece37634-bib-0015] Chazdon, R. L. (2003). Tropical forest recovery: Legacies of human impact and natural disturbances. Perspectives in Plant Ecology, Evolution and Systematics, 6(1–2), 51–71. 10.1078/1433-8319-00042

[ece37634-bib-0016] Chazdon, R. L. (2008). Chance and determinism in tropical forest succession. Tropical Forest Community Ecology, 10 32, 384–409.

[ece37634-bib-0017] Chesson, P. (2000). Mechanisms of maintenance of species diversity. Annual Review of Ecology and Systematics, 31(1), 343–366. 10.1146/annurev.ecolsys.31.1.343

[ece37634-bib-0018] Clark, J. S. , Macklin, E. , & Wood, L. (1998). Stages and spatial scales of recruitment limitation in Southern Appalachian forests. Ecological Monographs, 68(2), 213–235.

[ece37634-bib-0019] Clements, F. E. (1916). Plant Succession: An analysis of the development of vegetation (vol. 242). Carnegie Institution of Washington.

[ece37634-bib-0020] Coste, S. , Baraloto, C. , Leroy, C. , Marcon, É. , Renaud, A. , Richardson, A. D. , Roggy, J. C. , Schimann, H. , Uddling, J. , & Hérault, B. (2010). Assessing foliar chlorophyll contents with the SPAD‐502 chlorophyll meter: A calibration test with thirteen tree species of tropical rainforest in French Guiana. Annals of Forest Science, 67(6), 607. 10.1051/forest/2010020

[ece37634-bib-0021] Denslow, J. S. (1980). Gap partitioning among tropical rainforest trees. Biotropica, 12(2), 47–55. 10.2307/2388156

[ece37634-bib-0022] Denslow, J. S. , & Guzman, G. S. (2000). Variation in stand structure, light and seedling abundance across a tropical moist forest chronosequence, Panama. Journal of Vegetation Science, 11(2), 201–212. 10.2307/3236800

[ece37634-bib-0023] Dezécache, C. , Faure, E. , Gond, V. , Salles, J.‐M. , Vieilledent, G. , & Hérault, B. (2017). Gold‐rush in a forested El Dorado: Deforestation leakages and the need for regional cooperation. Environmental Research Letters, 12(3), 34013. 10.1088/1748-9326/aa6082

[ece37634-bib-0024] Dezécache, C. , Salles, J. M. , Vieilledent, G. , & Hérault, B. (2017). Moving forward socio‐economically focused models of deforestation. Global Change Biology, 23(9), 3484–3500. 10.1111/gcb.13611 28055134

[ece37634-bib-0025] Diaz, S. , Lavorel, S. , de Bello, F. , Quetier, F. , Grigulis, K. , & Robson, T. M. (2007). Incorporating plant functional diversity effects in ecosystem service assessments. Proceedings of the National Academy of Sciences of the United States of America, 104(52), 20684–20689. 10.1073/pnas.0704716104 18093933 PMC2410063

[ece37634-bib-0026] Díaz, S. , Stuart Chapin, III, F. , & Simon, P. (2005). Biodiversity regulation of ecosystem services. In Trends and conditions (pp. 297–329).

[ece37634-bib-0027] Fortunel, C. , Paine, C. E. , Fine, P. V. A. , Kraft, N. J. B. , & Baraloto, C. (2014). Environmental factors predict community functional composition in Amazonian Forests. Journal of Ecology, 102(1), 145–155. 10.1111/1365-2745.12160

[ece37634-bib-0028] Fukami, T. , Martijn Bezemer, T. , Mortimer, S. R. , & Van Der Putten, W. H. (2005). Species divergence and trait convergence in experimental plant community assembly. Ecology Letters, 8(12), 1283–1290. 10.1111/j.1461-0248.2005.00829.x

[ece37634-bib-0029] Gourlet‐Fleury, S. , Guehl, J. M. , & OIivier, L. (2004). Ecology & management of a neotropical rainforest. Lessons drawn from Paracou, a long‐term experimental research site in French Guiana. Elsevier.

[ece37634-bib-0030] Gourlet‐Fleury, S. , & Houllier, F. (2000). Modelling diameter increment in a lowland evergreen rain forest in French Guiana. Forest Ecology and Management, 131(1–3), 269–289. 10.1016/S0378-1127(99)00212-1

[ece37634-bib-0031] Grubb, P. J. (1977). The maintenance of species‐richness in plant communities: The importance of the regeneration Niche. Biological Reviews, 52(1), 107–145. 10.1111/j.1469-185X.1977.tb01347.x

[ece37634-bib-0032] Hérault, B. , Bachelot, B. , Poorter, L. , Rossi, V. , Bongers, F. , Jérôme Chave, C. E. , Paine, T. , Wagner, F. , & Baraloto, C. (2011). Functional traits shape ontogenetic growth trajectories of rain forest tree species. Journal of Ecology, 99, 1431–1440. 10.1111/j.1365-2745.2011.01883.x

[ece37634-bib-0033] Hérault, B. , & Piponiot, C. (2018). Key drivers of ecosystem recovery after disturbance in a Neotropical forest. Forest Ecosystems, 5(1), 2. 10.1186/s40663-017-0126-7

[ece37634-bib-0067] Hurtt, G. C. , & Pacala, S. W. (1995). The consequences of recruitment limitation: reconciling chance, history and competitive differences between plants. Journal of Theoretical Biology, 176(1), 1–12.

[ece37634-bib-0035] Jones, C. G. , Lawton, J. H. , & Shachak, M. (1994). Organisms as ecosystem engineers. In Ecosystem management (pp. 130–147). New York, NY: Springer.

[ece37634-bib-0036] Kunstler, G. , Falster, D. , Coomes, D. A. , Hui, F. , Kooyman, R. M. , Laughlin, D. C. , Poorter, L. , Vanderwel, M. , Vieilledent, G. , Wright, S. J. , Aiba, M. , Baraloto, C. , Caspersen, J. , Cornelissen, J. H. C. , Gourlet‐Fleury, S. , Hanewinkel, M. , Herault, B. , Kattge, J. , Kurokawa, H. , … Westoby, M. (2016). Plant functional traits have globally consistent effects on competition. Nature, 529(7585), 204–207. 10.1038/nature16476 26700807

[ece37634-bib-0037] Lawton, R. O. , & Putz, F. E. (1988). Natural disturbance and gap‐phase regeneration in a wind‐exposed tropical cloud forest. Ecology, 69(3), 764–777. 10.2307/1941025

[ece37634-bib-0038] Letcher, S. G. , Lasky, J. R. , Chazdon, R. L. , Norden, N. , Wright, S. J. , Meave, J. A. , Pérez‐García, E. A. , Muñoz, R. , Romero‐Pérez, E. , Andrade, A. , Andrade, J. L. , Balvanera, P. , Becknell, J. M. , Bentos, T. V. , Bhaskar, R. , Bongers, F. , Boukili, V. , Brancalion, P. H. S. , César, R. G. , … Williamson, G. B. (2015). Environmental gradients and the evolution of successional habitat specialization: A test case with 14 Neotropical Forest Sites. Journal of Ecology, 103(5), 1276–1290. 10.1111/1365-2745.12435

[ece37634-bib-0039] Longworth, J. B. , Mesquita, R. C. , Bentos, T. V. , Moreira, M. P. , Massoca, P. E. , & Williamson, G. B. (2014). Shifts in dominance and species assemblages over two decades in alternative successions in Central Amazonia. Biotropica, 46(5), 529–537. 10.1111/btp.12143

[ece37634-bib-0040] MacArthur, R. , & Levins, R. (1967). The limiting similarity, convergence, and divergence of coexisting species. The American Naturalist, 101(921), 377–385. 10.1086/282505

[ece37634-bib-0041] Magurran, A. E. (2004). Measuring biological diversity. Blackwell Science Ltd.

[ece37634-bib-0042] Marcon, E. , & Hérault, B. (2015). entropart: An R package to measure and partition diversity. Journal of Statistical Software, 67(8), 1–26.

[ece37634-bib-0043] Mason, N. W. H. , De Bello, F. , Mouillot, D. , Pavoine, S. , & Dray, S. (2013). A guide for using functional diversity indices to reveal changes in assembly processes along ecological gradients. Journal of Vegetation Science, 24(5), 794–806. 10.1111/jvs.12013

[ece37634-bib-0044] Meiners, S. J. , Cadotte, M. W. , Fridley, J. D. , Pickett, S. T. A. , & Walker, L. R. (2015). Is successional research nearing its climax? New approaches for understanding dynamic communities. Functional Ecology, 29(2), 154–164. 10.1111/1365-2435.12391

[ece37634-bib-0045] Mirabel, A. , Hérault, B. , & Marcon, E. (2020). Diverging taxonomic and functional trajectories following disturbance in a Neotropical forest. Science of the Total Environment, 720, 137397. 10.1016/j.scitotenv.2020.137397 32143035

[ece37634-bib-0046] Molino, J. F. , & Sabatier, D. (2001). Tree diversity in tropical rain forests: A validation of the intermediate disturbance hypothesis. Science, 294(5547), 1702–1704. 10.1126/science.1060284 11721052

[ece37634-bib-0047] Norden, N. , Angarita, H. A. , Bongers, F. , Martínez‐Ramos, M. , Granzow‐de la Cerda, I. , van Breugel, M. , Lebrija‐Trejos, E. , Meave, J. A. , Vandermeer, J. , Williamson, G. B. , Finegan, B. , Mesquita, R. , & Chazdon, R. L. (2015). Successional dynamics in Neotropical forests are as uncertain as they are predictable. Proceedings of the National Academy of Sciences of the United States of America, 112(26), 8013–8018. 10.1073/pnas.1500403112 26080411 PMC4491784

[ece37634-bib-0048] Ollivier, M. , Baraloto, C. , & Marcon, E. (2007). A Trait database for Guianan Rain Forest Trees permits intra‐and inter‐specific contrasts. Annals of Forest Science, 64(7), 781–786. 10.1051/forest:2007058

[ece37634-bib-0049] Paine, C. E. T. , Baraloto, C. , & Díaz, S. (2015). Optimal strategies for sampling functional traits in species‐rich forests. Functional Ecology, 29(10), 1325–1331. 10.1111/1365-2435.12433

[ece37634-bib-0050] Peet, R. K. (1992). Community structure and ecosystem function. Plant Succession: Theory and Prediction, 11, 103.

[ece37634-bib-0051] Peña‐Claros, M. , Peters, E. M. , Justiniano, M. J. , Bongers, F. J. J. M. , Blate, G. M. , Fredericksen, T. S. , & Putz, F. E. (2008). Regeneration of commercial tree species following Silvicultural Treatments in a Moist Tropical Forest. Forest Ecology and Management, 255(3–4), 1283–1293. 10.1016/j.foreco.2007.10.033

[ece37634-bib-0052] Perronne, R. , Munoz, F. , Borgy, B. , Reboud, X. , & Gaba, S. (2017). How to design trait‐based analyses of community assembly mechanisms: Insights and guidelines from a literature review. Perspectives in Plant Ecology, Evolution and Systematics, 25, 29–44. 10.1016/j.ppees.2017.01.004

[ece37634-bib-0053] Piponiot, C. , Sist, P. , Mazzei, L. , Peña‐Claros, M. , Putz, F. E. , Rutishauser, E. , Shenkin, A. , Ascarrunz, N. , de Azevedo, C. P. , Baraloto, C. , França, M. , Guedes, M. , Honorio Coronado, E. N. , d'Oliveira, M. V. N. , Ruschel, A. R. , da Silva, K. E. , Doff Sotta, E. , de Souza, C. R. , Vidal, E. , … Hérault, B. (2016). Carbon recovery dynamics following disturbance by selective logging in amazonian forests. eLife, 5, e21394. 10.7554/eLife.21394 27993185 PMC5217754

[ece37634-bib-0054] Podani, J. , Ricotta, C. , & Schmera, D. (2013). A general framework for analyzing beta diversity, nestedness and related community‐level phenomena based on abundance data. Ecological Complexity, 15, 52–61. 10.1016/j.ecocom.2013.03.002

[ece37634-bib-0055] Putz, F. E. , Zuidema, P. A. , Synnott, T. , Peña‐Claros, M. , Pinard, M. A. , Sheil, D. , Vanclay, J. K. , Sist, P. , Gourlet‐Fleury, S. , Griscom, B. , Palmer, J. , & Zagt, R. (2012). Sustaining conservation values in selectively logged tropical forests: The attained and the attainable. Conservation Letters, 5(4), 296–303. 10.1111/j.1755-263X.2012.00242.x

[ece37634-bib-0056] Rees, M. , Condit, R. , Crawley, M. , Pacala, S. , & Tilman, D. (2001). Long‐term studies of vegetation dynamics. Science, 293(5530), 650–655. 10.1126/science.1062586 11474101

[ece37634-bib-0057] Réjou‐Méchain, M. , Tanguy, A. , Piponiot, C. , Chave, J. , & Hérault, B. (2017). Biomass: An R package for estimating above‐ground biomass and its uncertainty in tropical forests. Methods in Ecology and Evolution, 8(9), 1163–1167.

[ece37634-bib-0058] Réjou‐Méchain, M. , Tanguy, A. , Piponiot, C. , Chave, J. , & Hérault, B. (2018). BIOMASS: Estimating aboveground biomass and its uncertainty in tropical forests. https://CRAN.R‐project.org/package=BIOMASS

[ece37634-bib-0059] Rutishauser, E. , Hérault, B. , Petronelli, P. , & Sist, P. (2016). Tree height reduction after selective logging in a tropical forest. Biotropica, 48(3), 285–289. 10.1111/btp.12326

[ece37634-bib-0060] Schleuning, M. , Fründ, J. , Schweiger, O. , Welk, E. , Albrecht, J. , Albrecht, M. , Beil, M. , Benadi, G. , Blüthgen, N. , Bruelheide, H. , Böhning‐Gaese, K. , Dehling, D. M. , Dormann, C. F. , Exeler, N. , Farwig, N. , Harpke, A. , Hickler, T. , Kratochwil, A. , Kuhlmann, M. , … Hof, C. (2016). Ecological networks are more sensitive to plant than to animal extinction under climate change. Nature Communications, 7, 13965. 10.1038/ncomms13965 PMC5196430 28008919

[ece37634-bib-0061] Svenning, J.‐C. , & Wright, S. J. (2005). Seed limitation in a Panamanian Forest. Journal of Ecology, 93(5), 853–862. 10.1111/j.1365-2745.2005.01016.x

[ece37634-bib-0062] van Buuren, S. , & Groothuis‐Oudshoorn, K. (2011). mice: Multivariate imputation by chained equations in R. Journal of Statistical Software, 45(3), 1–67.

[ece37634-bib-0063] Violle, C. , Navas, M.‐L. , Vile, D. , Kazakou, E. , Fortunel, C. , Hummel, I. , & Garnier, É. (2007). Let the concept of trait be functional!. Oikos, 116(5), 882–892. 10.1111/j.2007.0030-1299.15559.x

[ece37634-bib-0064] Wagner, F. , Hérault, B. , Stahl, C. , Bonal, D. , & Rossi, V. (2011). Modeling water availability for trees in tropical forests. Agricultural and Forest Meteorology, 151(9), 1202–1213. 10.1016/j.agrformet.2011.04.012

[ece37634-bib-0065] Wood, S. N. (2011). Fast stable restricted Maximum Likelihood and Marginal Likelihood estimation of semiparametric Generalized Linear Models. Journal of the Royal Statistical Society: Series B (Statistical Methodology), 73(1), 3–36. 10.1111/j.1467-9868.2010.00749.x

[ece37634-bib-0066] Wright, I. J. , Reich, P. B. , Westoby, M. , Ackerly, D. D. , Baruch, Z. , Bongers, F. , Cavender‐Bares, J. , Chapin, T. , Cornelissen, J. H. C. , Diemer, M. , Flexas, J. , Garnier, E. , Groom, P. K. , Gulias, J. , Hikosaka, K. , Lamont, B. B. , Lee, T. , Lee, W. , Lusk, C. , … Villar, R. (2004). The worldwide leaf economics spectrum. Nature, 428(6985), 821–827. 10.1038/nature02403 15103368

