# Supplementary material for: 30 Years of postdisturbance recruitment in a Neotropical forest
Source: Ecol Evol. 2021 Oct 7;11(21):14448–58. doi: 10.1002/ece3.7634 (PMC8571577; doi:10.1002/ece3.7634)
Supplement: Supplementary file 1 — Appendix S1 [file ECE3-11-14448-s001.docx]

30 YEARS OF POST-DISTURBANCE RECRUITMENT IN A NEOTROPICAL FOREST

APPENDIX 1

Mirabel Ariane^1^, Marcon Eric^1^, Hérault Bruno^2, 3, 4^

1 UMR EcoFoG, AgroParistech, CNRS, Cirad, INRA, Université des Antilles, Université de Guyane.

2 CIRAD, UPR Forêts et Sociétés, Yamoussoukro, Côte d’Ivoire.

3 Forêts et Sociétés, Univ Montpellier, CIRAD, Montpellier, France

4 Institut National Polytechnique Félix Houphouët-Boigny, INP-HB, Yamoussoukro, Côte d’Ivoire.

Correspondence:

*Ariane Mirabel*

*Email:* [Ariane.Mirabel@gmail.com](mailto:Ariane.Mirabel@ecofog.gf).

**Appendix S1:** Pearson correlation coefficients computed from species traits for the seven functional traits and life-history trait considered.

|  | Leaf thickness | Leaf chlorophyll content | Leaf toughness | Bark thickness | SLA | WD | Hmax |
| --- | --- | --- | --- | --- | --- | --- | --- |
| Leaf thickness | 1 | 0.33 | 0.56 | 0.36 | 0.18 | -0.12 | 0.05 |
| Leaf chlorophyll content |  | 1 | 0.24 | -0.04 | -0.04 | -0.05 | -0.06 |
| Leaf toughness |  |  | 1 | 0.37 | 0.24 | 0.09 | -0.03 |
| Bark thickness |  |  |  | 1 | 0.84 | -0.23 | -0.11 |
| SLA |  |  |  |  | 1 | -0.23 | -0.19 |
| WD |  |  |  |  |  | 1 | 0 |
| Hmax |  |  |  |  |  |  | 1 |
