# Supplementary material for: 30 Years of postdisturbance recruitment in a Neotropical forest
Source: Ecol Evol. 2021 Oct 7;11(21):14448–58. doi: 10.1002/ece3.7634 (PMC8571577; doi:10.1002/ece3.7634)
Supplement: Supplementary file 2 — Appendix S2 [file ECE3-11-14448-s002.docx]

30 YEARS OF POST-DISTURBANCE RECRUITMENT IN A NEOTROPICAL FOREST

APPENDIX 2

Mirabel Ariane^1^, Marcon Eric^1^, Hérault Bruno^2, 3, 4^

1 UMR EcoFoG, AgroParistech, CNRS, Cirad, INRA, Université des Antilles, Université de Guyane.

2 CIRAD, UPR Forêts et Sociétés, Yamoussoukro, Côte d’Ivoire.

3 Forêts et Sociétés, Univ Montpellier, CIRAD, Montpellier, France

4 Institut National Polytechnique Félix Houphouët-Boigny, INP-HB, Yamoussoukro, Côte d’Ivoire.

Correspondence:

*Ariane Mirabel*

*Email:* [Ariane.Mirabel@gmail.com](mailto:Ariane.Mirabel@ecofog.gf).


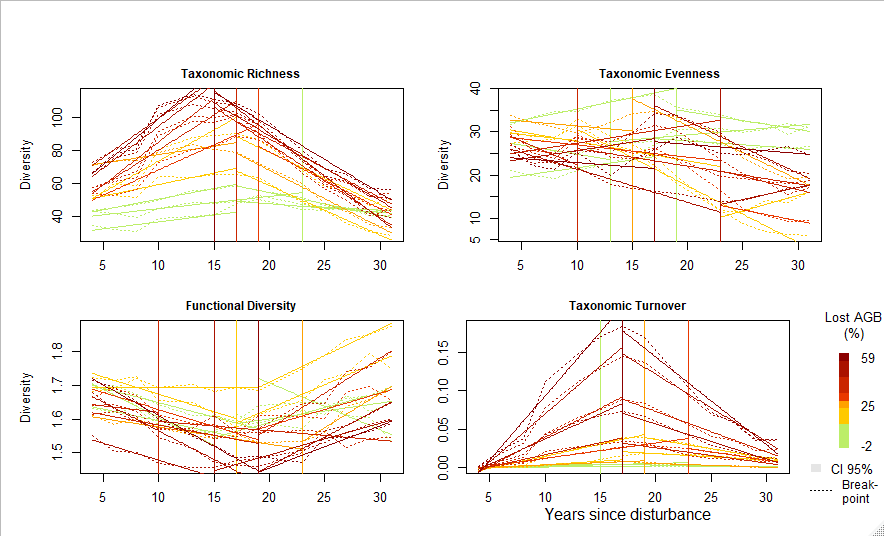
**Appendix S2:** Breakpoints analysis of post-disturbance trajectories regarding, from left to right, taxonomic richness, taxonomic evenness, functional diversity, and taxonomic turnover of 2-years laps recruited communities. The best linear models segmented according to break points are selected based on their mean square errors. Dots are the observed trajectories, plain lines are linear model, and vertical dotted lines are the break points. Lines color correspond to initial disturbance intensity in percentage of AGB lost.
