# Supplementary material for: 30 Years of postdisturbance recruitment in a Neotropical forest
Source: Ecol Evol. 2021 Oct 7;11(21):14448–58. doi: 10.1002/ece3.7634 (PMC8571577; doi:10.1002/ece3.7634)
Supplement: Supplementary file 3 — Appendix S3 [file ECE3-11-14448-s003.docx]

30 YEARS OF POST-DISTURBANCE RECRUITMENT IN A NEOTROPICAL FOREST

APPENDIX 3

Mirabel A.^1^, Marcon E.^1^, Hérault B.^2, 3, 4^

1 UMR EcoFoG, AgroParistech, CNRS, Cirad, INRA, Université des Antilles, Université de Guyane.

2 CIRAD, UPR Forêts et Sociétés, Yamoussoukro, Côte d’Ivoire.

3 Forêts et Sociétés, Univ Montpellier, CIRAD, Montpellier, France

4 Institut National Polytechnique Félix Houphouët-Boigny, INP-HB, Yamoussoukro, Côte d’Ivoire.

Correspondence:

*Ariane Mirabel*

*Email:* [Ariane.Mirabel@g](mailto:Ariane.Mirabel@ecofog.gf)mail.com

Appendix S3: List of recruited species for all plots throughout the 30 years inventoried.

| **Family** | **Genus** | **Species** |
| --- | --- | --- |
| Anacardiaceae | *Anacardium* | *spruceanum* |
| Anacardiaceae | *Tapirira* | *bethanniana* |
| Anacardiaceae | *Tapirira* | *guianensis* |
| Anacardiaceae | *Tapirira* | *obtusa* |
| Anacardiaceae | *Thyrsodium* | *guianense* |
| Anacardiaceae | *Thyrsodium* | *puberulum* |
| Anacardiaceae | *Thyrsodium* | *spruceanum* |
| Annonaceae | *Anaxagorea* | *acuminata* |
| Annonaceae | *Anaxagorea* | *dolichocarpa* |
| Annonaceae | *Annona* | *ambotay* |
| Annonaceae | *Annona* | *exsucca* |
| Annonaceae | *Annona* | *foetida* |
| Annonaceae | *Annona* | *prevostiae* |
| Annonaceae | *Duguetia* | *calycina* |
| Annonaceae | *Duguetia* | *yeshidan* |
| Annonaceae | *Fusaea* | *longifolia* |
| Annonaceae | *Guatteria* | *citriodora* |
| Annonaceae | *Guatteria* | *guianensis* |
| Annonaceae | *Guatteria* | *punctata* |
| Annonaceae | *Guatteria* | *schomburgkiana* |
| Annonaceae | *Oxandra* | *asbeckii* |
| Annonaceae | *Unonopsis* | *rufescens* |
| Annonaceae | *Xylopia* | *aromatica* |
| Annonaceae | *Xylopia* | *cayennensis* |
| Annonaceae | *Xylopia* | *crinita* |
| Annonaceae | *Xylopia* | *frutescens* |
| Annonaceae | *Xylopia* | *nitida* |
| Annonaceae | *Xylopia* | *pulcherrima* |
| Annonaceae | *Xylopia* | *surinamensis* |
| Apocynaceae | *Ambelania* | *acida* |
| Apocynaceae | *Aspidosperma* | *album* |
| Apocynaceae | *Aspidosperma* | *desmanthum* |
| Apocynaceae | *Aspidosperma* | *excelsum* |
| Apocynaceae | *Aspidosperma* | *helstonei* |
| Apocynaceae | *Aspidosperma* | *oblongum* |
| Apocynaceae | *Aspidosperma* | *spruceanum* |
| Apocynaceae | *Couma* | *guianensis* |
| Apocynaceae | *Himatanthus* | *articulatus* |
| Apocynaceae | *Himatanthus* | *bracteatus* |
| Apocynaceae | *Lacmellea* | *aculeata* |
| Apocynaceae | *Macoubea* | *guianensis* |
| Apocynaceae | *Parahancornia* | *fasciculata* |
| Apocynaceae | *Rauvolfia* | *paraensis* |
| Apocynaceae | *Tabernaemontana* | *attenuata* |
| Aquifoliaceae | *Ilex* | *inundata* |
| Aquifoliaceae | *Ilex* | *sp.2CAY-ATDN* |
| Araliaceae | *Schefflera* | *decaphylla* |
| Arecaceae | *Astrocaryum* | *sciophilum* |
| Arecaceae | *Attalea* | *maripa* |
| Arecaceae | *Euterpe* | *oleracea* |
| Arecaceae | *Oenocarpus* | *bacaba* |
| Arecaceae | *Oenocarpus* | *bataua* |
| Arecaceae | *Socratea* | *exorrhiza* |
| Arecaceae | *Syagrus* | *inajai* |
| Bignoniaceae | *Handroanthus* | *serratifolius* |
| Bignoniaceae | *Jacaranda* | *copaia* |
| Bignoniaceae | *Tabebuia* | *insignis* |
| Boraginaceae | *Cordia* | *exaltata* |
| Boraginaceae | *Cordia* | *goeldiana* |
| Boraginaceae | *Cordia* | *nervosa* |
| Boraginaceae | *Cordia* | *panicularis* |
| Boraginaceae | *Cordia* | *sagotii* |
| Boraginaceae | *Cordia* | *sprucei* |
| Boraginaceae | *Cordia* | *toqueve* |
| Burseraceae | *Dacryodes* | *nitens* |
| Burseraceae | *Dacryodes* | *sp.4CAY-ATDN* |
| Burseraceae | *Protium* | *apiculatum* |
| Burseraceae | *Protium* | *decandrum* |
| Burseraceae | *Protium* | *gallicum* |
| Burseraceae | *Protium* | *giganteum* |
| Burseraceae | *Protium* | *guianense* |
| Burseraceae | *Protium* | *opacum* |
| Burseraceae | *Protium* | *plagiocarpium* |
| Burseraceae | *Protium* | *sagotianum* |
| Burseraceae | *Protium* | *subserratum* |
| Burseraceae | *Protium* | *tenuifolium* |
| Burseraceae | *Protium* | *trifoliolatum* |
| Burseraceae | *Tetragastris* | *altissima* |
| Burseraceae | *Tetragastris* | *hostmannii* |
| Burseraceae | *Tetragastris* | *panamensis* |
| Burseraceae | *Trattinnickia* | *burserifolia* |
| Burseraceae | *Trattinnickia* | *demerarae* |
| Burseraceae | *Trattinnickia* | *rhoifolia* |
| Calophyllaceae | *Caraipa* | *densifolia* |
| Calophyllaceae | *Caraipa* | *racemosa* |
| Calophyllaceae | *Mahurea* | *palustris* |
| Capparaceae | *Capparidastrum* | *frondosum* |
| Capparaceae | *Neocalyptrocalyx* | *leprieurii* |
| Cardiopteridaceae | *Dendrobangia* | *boliviana* |
| Caryocaraceae | *Caryocar* | *glabrum* |
| Caryocaraceae | *Caryocar* | *microcarpum* |
| Celastraceae | *Cheiloclinium* | *cognatum* |
| Celastraceae | *Hippocratea* | *volubilis* |
| Celastraceae | *Maytenus* | *guyanensis* |
| Celastraceae | *Maytenus* | *oblongata* |
| Celastraceae | *Maytenus* | *sp.7CAY-ATDN* |
| Chrysobalanaceae | *Couepia* | *bracteosa* |
| Chrysobalanaceae | *Couepia* | *caryophylloides* |
| Chrysobalanaceae | *Couepia* | *guianensis* |
| Chrysobalanaceae | *Couepia* | *habrantha* |
| Chrysobalanaceae | *Couepia* | *magnoliifolia* |
| Chrysobalanaceae | *Couepia* | *obovata* |
| Chrysobalanaceae | *Couepia* | *parillo* |
| Chrysobalanaceae | *Gaulettia* | *parillo* |
| Chrysobalanaceae | *Hirtella* | *bicornis* |
| Chrysobalanaceae | *Hirtella* | *glandulosa* |
| Chrysobalanaceae | *Hirtella* | *hispidula* |
| Chrysobalanaceae | *Licania* | *alba* |
| Chrysobalanaceae | *Licania* | *canescens* |
| Chrysobalanaceae | *Licania* | *densiflora* |
| Chrysobalanaceae | *Licania* | *granvillei* |
| Chrysobalanaceae | *Licania* | *heteromorpha* |
| Chrysobalanaceae | *Licania* | *hypoleuca* |
| Chrysobalanaceae | *Licania* | *latifolia* |
| Chrysobalanaceae | *Licania* | *latistipula* |
| Chrysobalanaceae | *Licania* | *laxiflora* |
| Chrysobalanaceae | *Licania* | *licaniiflora* |
| Chrysobalanaceae | *Licania* | *longistyla* |
| Chrysobalanaceae | *Licania* | *majuscula* |
| Chrysobalanaceae | *Licania* | *membranacea* |
| Chrysobalanaceae | *Licania* | *micrantha* |
| Chrysobalanaceae | *Licania* | *ovalifolia* |
| Chrysobalanaceae | *Licania* | *parvifructa* |
| Chrysobalanaceae | *Licania* | *robusta* |
| Chrysobalanaceae | *Licania* | *sprucei* |
| Chrysobalanaceae | *Parinari* | *campestris* |
| Chrysobalanaceae | *Parinari* | *montana* |
| Chrysobalanaceae | *Parinari* | *parvifolia* |
| Chrysobalanaceae | *Parinari* | *rodolphii* |
| Chrysobalanaceae | *Indet.* | *sp.1CAY-ATDN* |
| Clusiaceae | *Clusia* | *grandiflora* |
| Clusiaceae | *Garcinia* | *benthamiana* |
| Clusiaceae | *Garcinia* | *madruno* |
| Clusiaceae | *Moronobea* | *coccinea* |
| Clusiaceae | *Platonia* | *insignis* |
| Clusiaceae | *Symphonia* | *globulifera* |
| Clusiaceae | *Symphonia* | *sp.1* |
| Clusiaceae | *Tovomita* | *brevistaminea* |
| Clusiaceae | *Tovomita* | *macrophylla* |
| Clusiaceae | *Tovomita* | *obovata* |
| Clusiaceae | *Tovomita* | *sp.1* |
| Clusiaceae | *Tovomita* | *sp.10CAY-ATDN* |
| Clusiaceae | *Tovomita* | *sp.11CAY-ATDN* |
| Clusiaceae | *Tovomita* | *sp.2* |
| Clusiaceae | *Tovomita* | *sp.22CAY-ATDN* |
| Clusiaceae | *Tovomita* | *sp.2CAY-ATDN* |
| Clusiaceae | *Tovomita* | *sp.3CAY-ATDN* |
| Clusiaceae | *Tovomita* | *sp.5CAY-ATDN* |
| Clusiaceae | *Tovomita* | *sp.9CAY-ATDN* |
| Clusiaceae | *Tovomita* | *sp.B1* |
| Combretaceae | *Buchenavia* | *grandis* |
| Combretaceae | *Buchenavia* | *guianensis* |
| Combretaceae | *Buchenavia* | *nitidissima* |
| Combretaceae | *Buchenavia* | *tetraphylla* |
| Combretaceae | *Terminalia* | *amazonia* |
| Convolvulaceae | *Dicranostyles* | *integra* |
| Dichapetalaceae | *Tapura* | *amazonica* |
| Dichapetalaceae | *Tapura* | *capitulifera* |
| Dilleniaceae | *Doliocarpus* | *sp.1* |
| Ebenaceae | *Diospyros* | *capreifolia* |
| Ebenaceae | *Diospyros* | *carbonaria* |
| Ebenaceae | *Diospyros* | *guianensis* |
| Ebenaceae | *Diospyros* | *vestita* |
| Elaeocarpaceae | *Sloanea* | *brevipes* |
| Elaeocarpaceae | *Sloanea* | *garckeana* |
| Elaeocarpaceae | *Sloanea* | *grandiflora* |
| Elaeocarpaceae | *Sloanea* | *guianensis* |
| Elaeocarpaceae | *Sloanea* | *latifolia* |
| Elaeocarpaceae | *Sloanea* | *latifolia_form2* |
| Elaeocarpaceae | *Sloanea* | *laxiflora* |
| Elaeocarpaceae | *Sloanea* | *parviflora* |
| Elaeocarpaceae | *Sloanea* | *sinemariensis* |
| Elaeocarpaceae | *Sloanea* | *sp.1* |
| Elaeocarpaceae | *Sloanea* | *sp.14CAY-ATDN* |
| Elaeocarpaceae | *Sloanea* | *sp.17CAY-ATDN* |
| Elaeocarpaceae | *Sloanea* | *sp.20CAY-ATDN* |
| Elaeocarpaceae | *Sloanea* | *sp.21CAY-ATDN* |
| Elaeocarpaceae | *Sloanea* | *sp.22CAY-ATDN* |
| Elaeocarpaceae | *Sloanea* | *sp.24CAY-ATDN* |
| Elaeocarpaceae | *Sloanea* | *sp.2CAY-ATDN* |
| Elaeocarpaceae | *Sloanea* | *sp.4CAY-ATDN* |
| Elaeocarpaceae | *Sloanea* | *sp.5CAY-ATDN* |
| Elaeocarpaceae | *Sloanea* | *sp.8CAY-ATDN* |
| Elaeocarpaceae | *Sloanea* | *sp.P33* |
| Elaeocarpaceae | *Sloanea* | *tuerckheimii* |
| Emmotaceae | *Emmotum* | *fagifolium* |
| Erythroxylaceae | *Erythroxylum* | *citrifolium* |
| Erythroxylaceae | *Erythroxylum* | *ligustrinum* |
| Erythroxylaceae | *Erythroxylum* | *lineolatum* |
| Erythroxylaceae | *Erythroxylum* | *sp.1CAY-ATDN* |
| Euphorbiaceae | *Alchornea* | *discolor* |
| Euphorbiaceae | *Alchornea* | *triplinervia* |
| Euphorbiaceae | *Alchorneopsis* | *floribunda* |
| Euphorbiaceae | *Chaetocarpus* | *schomburgkianus* |
| Euphorbiaceae | *Chaetocarpus* | *sp.1CAY-ATDN* |
| Euphorbiaceae | *Conceveiba* | *guianensis* |
| Euphorbiaceae | *Glycydendron* | *amazonicum* |
| Euphorbiaceae | *Hevea* | *guianensis* |
| Euphorbiaceae | *Mabea* | *piriri* |
| Euphorbiaceae | *Pera* | *glabrata* |
| Euphorbiaceae | *Pogonophora* | *schomburgkiana* |
| Euphorbiaceae | *Sagotia* | *racemosa* |
| Euphorbiaceae | *Sandwithia* | *guyanensis* |
| Euphorbiaceae | *Indet.* | *sp.P4* |
| Fabaceae | *Abarema* | *jupunba* |
| Fabaceae | *Abarema* | *mataybifolia* |
| Fabaceae | *Albizia* | *pedicellaris* |
| Fabaceae | *Alexa* | *wachenheimii* |
| Fabaceae | *Andira* | *coriacea* |
| Fabaceae | *Bocoa* | *prouacensis* |
| Fabaceae | *Cassia* | *spruceana* |
| Fabaceae | *Copaifera* | *guianensis* |
| Fabaceae | *Dialium* | *guianense* |
| Fabaceae | *Dicorynia* | *guianensis* |
| Fabaceae | *Dimorphandra* | *polyandra* |
| Fabaceae | *Diplotropis* | *purpurea* |
| Fabaceae | *Dipteryx* | *odorata* |
| Fabaceae | *Enterolobium* | *oldemanii* |
| Fabaceae | *Enterolobium* | *schomburgkii* |
| Fabaceae | *Enterolobium* | *sp.1CAY-ATDN* |
| Fabaceae | *Eperua* | *falcata* |
| Fabaceae | *Eperua* | *grandiflora* |
| Fabaceae | *Eperua* | *rubiginosa* |
| Fabaceae | *Hymenolobium* | *flavum* |
| Fabaceae | *Inga* | *acreana* |
| Fabaceae | *Inga* | *acrocephala* |
| Fabaceae | *Inga* | *alba* |
| Fabaceae | *Inga* | *brachystachys* |
| Fabaceae | *Inga* | *brevipes* |
| Fabaceae | *Inga* | *capitata* |
| Fabaceae | *Inga* | *capitata_form2* |
| Fabaceae | *Inga* | *cayennensis* |
| Fabaceae | *Inga* | *cordatoalata* |
| Fabaceae | *Inga* | *cylindrica* |
| Fabaceae | *Inga* | *disticha* |
| Fabaceae | *Inga* | *fanchoniana* |
| Fabaceae | *Inga* | *graciliflora* |
| Fabaceae | *Inga* | *gracilifolia* |
| Fabaceae | *Inga* | *jenmanii* |
| Fabaceae | *Inga* | *lomatophylla* |
| Fabaceae | *Inga* | *longipedunculata* |
| Fabaceae | *Inga* | *loubryana* |
| Fabaceae | *Inga* | *marginata* |
| Fabaceae | *Inga* | *melinonis* |
| Fabaceae | *Inga* | *nobilis* |
| Fabaceae | *Inga* | *nouragensis* |
| Fabaceae | *Inga* | *paraensis* |
| Fabaceae | *Inga* | *pezizifera* |
| Fabaceae | *Inga* | *rubiginosa* |
| Fabaceae | *Inga* | *sarmentosa* |
| Fabaceae | *Inga* | *sp.12CAY-ATDN* |
| Fabaceae | *Inga* | *sp.16CAY-ATDN* |
| Fabaceae | *Inga* | *sp.18CAY-ATDN* |
| Fabaceae | *Inga* | *sp.4* |
| Fabaceae | *Inga* | *splendens* |
| Fabaceae | *Inga* | *stipularis* |
| Fabaceae | *Inga* | *thibaudiana* |
| Fabaceae | *Inga* | *tubiformis* |
| Fabaceae | *Inga* | *umbellifera* |
| Fabaceae | *Inga* | *virgultosa* |
| Fabaceae | *Macrolobium* | *bifolium* |
| Fabaceae | *Mimosa* | *guilandinae* |
| Fabaceae | *Ormosia* | *bolivarensis* |
| Fabaceae | *Ormosia* | *coccinea* |
| Fabaceae | *Ormosia* | *coutinhoi* |
| Fabaceae | *Ormosia* | *melanocarpa* |
| Fabaceae | *Ormosia* | *paraensis* |
| Fabaceae | *Ormosia* | *stipularis* |
| Fabaceae | *Parkia* | *gigantocarpa* |
| Fabaceae | *Parkia* | *nitida* |
| Fabaceae | *Parkia* | *pendula* |
| Fabaceae | *Parkia* | *ulei* |
| Fabaceae | *Parkia* | *velutina* |
| Fabaceae | *Peltogyne* | *paniculata* |
| Fabaceae | *Peltogyne* | *sp.1CAY-ATDN* |
| Fabaceae | *Peltogyne* | *sp.2CAY-ATDN* |
| Fabaceae | *Platymiscium* | *pinnatum* |
| Fabaceae | *Poecilanthe* | *effusa* |
| Fabaceae | *Poecilanthe* | *hostmannii* |
| Fabaceae | *Pseudopiptadenia* | *psilostachya* |
| Fabaceae | *Pterocarpus* | *officinalis* |
| Fabaceae | *Pterocarpus* | *rohrii* |
| Fabaceae | *Recordoxylon* | *speciosum* |
| Fabaceae | *Stryphnodendron* | *polystachyum* |
| Fabaceae | *Stryphnodendron* | *sp.3CAY-ATDN* |
| Fabaceae | *Swartzia* | *arborescens* |
| Fabaceae | *Swartzia* | *benthamiana* |
| Fabaceae | *Swartzia* | *grandifolia* |
| Fabaceae | *Swartzia* | *guianensis* |
| Fabaceae | *Swartzia* | *leblondii* |
| Fabaceae | *Swartzia* | *oblanceolata* |
| Fabaceae | *Swartzia* | *panacoco* |
| Fabaceae | *Swartzia* | *polyphylla* |
| Fabaceae | *Tachigali* | *guianensis* |
| Fabaceae | *Tachigali* | *melinonii* |
| Fabaceae | *Tachigali* | *paraensis* |
| Fabaceae | *Tachigali* | *richardiana* |
| Fabaceae | *Tachigali* | *sp.1* |
| Fabaceae | *Tachigali* | *sp.5CAY-ATDN* |
| Fabaceae | *Vatairea* | *erythrocarpa* |
| Fabaceae | *Vatairea* | *paraensis* |
| Fabaceae | *Vataireopsis* | *surinamensis* |
| Fabaceae | *Vouacapoua* | *americana* |
| Fabaceae | *Zygia* | *tetragona* |
| Goupiaceae | *Goupia* | *glabra* |
| Humiriaceae | *Humiria* | *balsamifera* |
| Humiriaceae | *Humiriastrum* | *excelsum* |
| Humiriaceae | *Humiriastrum* | *subcrenatum* |
| Humiriaceae | *Sacoglottis* | *cydonioides* |
| Humiriaceae | *Sacoglottis* | *guianensis* |
| Humiriaceae | *Vantanea* | *guianensis* |
| Humiriaceae | *Vantanea* | *parviflora* |
| Hypericaceae | *Vismia* | *cayennensis* |
| Hypericaceae | *Vismia* | *guianensis* |
| Hypericaceae | *Vismia* | *latifolia* |
| Hypericaceae | *Vismia* | *ramuliflora* |
| Hypericaceae | *Vismia* | *sessilifolia* |
| Hypericaceae | *Vismia* | *sp.1Guyafor* |
| Hypericaceae | *Vismia* | *sp.P1* |
| Icacinaceae | *Poraqueiba* | *guianensis* |
| Lacistemataceae | *Lacistema* | *aggregatum* |
| Lacistemataceae | *Lacistema* | *grandifolium* |
| Lacistemataceae | *Lacistema* | *polystachyum* |
| Lamiaceae | *Vitex* | *guianensis* |
| Lamiaceae | *Vitex* | *triflora* |
| Lauraceae | *Aniba* | *citrifolia* |
| Lauraceae | *Aniba* | *guianensis* |
| Lauraceae | *Aniba* | *rosaeodora* |
| Lauraceae | *Aniba* | *taubertiana* |
| Lauraceae | *Aniba* | *williamsii* |
| Lauraceae | *Endlicheria* | *melinonii* |
| Lauraceae | *Licaria* | *cannella* |
| Lauraceae | *Licaria* | *chrysophylla* |
| Lauraceae | *Licaria* | *debilis* |
| Lauraceae | *Licaria* | *guianensis* |
| Lauraceae | *Licaria* | *martiniana* |
| Lauraceae | *Mezilaurus* | *sp.1CAY-ATDN* |
| Lauraceae | *Nectandra* | *globosa* |
| Lauraceae | *Ocotea* | *amazonica* |
| Lauraceae | *Ocotea* | *argyrophylla* |
| Lauraceae | *Ocotea* | *cernua* |
| Lauraceae | *Ocotea* | *cinerea* |
| Lauraceae | *Ocotea* | *glomerata* |
| Lauraceae | *Ocotea* | *nigra* |
| Lauraceae | *Ocotea* | *oblonga* |
| Lauraceae | *Ocotea* | *percurrens* |
| Lauraceae | *Ocotea* | *puberula* |
| Lauraceae | *Ocotea* | *splendens* |
| Lauraceae | *Ocotea* | *subterminalis* |
| Lauraceae | *Ocotea* | *tomentella* |
| Lauraceae | *Rhodostemonodaphne* | *grandis* |
| Lauraceae | *Rhodostemonodaphne* | *kunthiana* |
| Lauraceae | *Rhodostemonodaphne* | *morii* |
| Lauraceae | *Rhodostemonodaphne* | *rufovirgata* |
| Lauraceae | *Sextonia* | *rubra* |
| Lauraceae | *Indet.* | *sp.30CAY-ATDN* |
| Lauraceae | *Indet.* | *sp.34CAY-ATDN* |
| Lauraceae | *Indet.* | *sp.38Guyafor* |
| Lauraceae | *Indet.* | *sp.39Guyafor* |
| Lauraceae | *Indet.* | *sp.B7* |
| Lecythidaceae | *Couratari* | *calycina* |
| Lecythidaceae | *Couratari* | *gloriosa* |
| Lecythidaceae | *Couratari* | *guianensis* |
| Lecythidaceae | *Couratari* | *multiflora* |
| Lecythidaceae | *Couratari* | *oblongifolia* |
| Lecythidaceae | *Eschweilera* | *collina* |
| Lecythidaceae | *Eschweilera* | *congestiflora* |
| Lecythidaceae | *Eschweilera* | *coriacea* |
| Lecythidaceae | *Eschweilera* | *decolorans* |
| Lecythidaceae | *Eschweilera* | *grandiflora* |
| Lecythidaceae | *Eschweilera* | *grandiflora_form2* |
| Lecythidaceae | *Eschweilera* | *parviflora* |
| Lecythidaceae | *Eschweilera* | *pedicellata* |
| Lecythidaceae | *Eschweilera* | *sagotiana* |
| Lecythidaceae | *Eschweilera* | *simiorum* |
| Lecythidaceae | *Eschweilera* | *wachenheimii* |
| Lecythidaceae | *Gustavia* | *augusta* |
| Lecythidaceae | *Gustavia* | *hexapetala* |
| Lecythidaceae | *Lecythis* | *chartacea* |
| Lecythidaceae | *Lecythis* | *corrugata* |
| Lecythidaceae | *Lecythis* | *corrugata subsp. corrugata* |
| Lecythidaceae | *Lecythis* | *holcogyne* |
| Lecythidaceae | *Lecythis* | *idatimon* |
| Lecythidaceae | *Lecythis* | *persistens* |
| Lecythidaceae | *Lecythis* | *poiteaui* |
| Lecythidaceae | *Lecythis* | *zabucajo* |
| Lecythidaceae | *Indet.* | *sp.6Guyafor* |
| Lecythidaceae | *Indet.* | *sp.7Guyafor* |
| Lecythidaceae | *Indet.* | *sp.8Guyafor* |
| Linaceae | *Hebepetalum* | *humiriifolium* |
| Loganiaceae | *Antonia* | *ovata* |
| Malpighiaceae | *Byrsonima* | *aerugo* |
| Malpighiaceae | *Byrsonima* | *densa* |
| Malpighiaceae | *Byrsonima* | *laevigata* |
| Malvaceae | *Apeiba* | *glabra* |
| Malvaceae | *Apeiba* | *petoumo* |
| Malvaceae | *Catostemma* | *fragrans* |
| Malvaceae | *Eriotheca* | *globosa* |
| Malvaceae | *Eriotheca* | *longitubulosa* |
| Malvaceae | *Luehea* | *speciosa* |
| Malvaceae | *Lueheopsis* | *rugosa* |
| Malvaceae | *Pachira* | *dolichocalyx* |
| Malvaceae | *Pachira* | *insignis* |
| Malvaceae | *Sterculia* | *excelsa* |
| Malvaceae | *Sterculia* | *multiovula* |
| Malvaceae | *Sterculia* | *pruriens* |
| Malvaceae | *Sterculia* | *sp.P1* |
| Malvaceae | *Sterculia* | *speciosa* |
| Malvaceae | *Theobroma* | *subincanum* |
| Malvaceae | *Theobroma* | *velutinum* |
| Melastomataceae | *Bellucia* | *grossularioides* |
| Melastomataceae | *Henriettea* | *succosa* |
| Melastomataceae | *Henriettella* | *flavescens* |
| Melastomataceae | *Loreya* | *arborescens* |
| Melastomataceae | *Loreya* | *mespiloides* |
| Melastomataceae | *Miconia* | *acuminata* |
| Melastomataceae | *Miconia* | *argyrophylla* |
| Melastomataceae | *Miconia* | *fragilis* |
| Melastomataceae | *Miconia* | *hypoleuca* |
| Melastomataceae | *Miconia* | *minutiflora* |
| Melastomataceae | *Miconia* | *plukenetii* |
| Melastomataceae | *Miconia* | *poeppigii* |
| Melastomataceae | *Miconia* | *prasina* |
| Melastomataceae | *Miconia* | *ruficalyx* |
| Melastomataceae | *Miconia* | *trinervia* |
| Melastomataceae | *Miconia* | *tschudyoides* |
| Melastomataceae | *Mouriri* | *angulicosta* |
| Melastomataceae | *Mouriri* | *crassifolia* |
| Melastomataceae | *Mouriri* | *dumetosa* |
| Melastomataceae | *Mouriri* | *huberi* |
| Melastomataceae | *Mouriri* | *nervosa* |
| Melastomataceae | *Mouriri* | *sagotiana* |
| Melastomataceae | *Mouriri* | *sp.2CAY-ATDN* |
| Melastomataceae | *Votomita* | *guianensis* |
| Meliaceae | *Carapa* | *procera* |
| Meliaceae | *Carapa* | *surinamensis* |
| Meliaceae | *Guarea* | *carinata* |
| Meliaceae | *Guarea* | *costata* |
| Meliaceae | *Guarea* | *glabra* |
| Meliaceae | *Guarea* | *kunthiana* |
| Meliaceae | *Guarea* | *pubescens* |
| Meliaceae | *Trichilia* | *cipo* |
| Meliaceae | *Trichilia* | *micrantha* |
| Meliaceae | *Trichilia* | *schomburgkii* |
| Moraceae | *Bagassa* | *guianensis* |
| Moraceae | *Brosimum* | *acutifolium* |
| Moraceae | *Brosimum* | *guianense* |
| Moraceae | *Brosimum* | *rubescens* |
| Moraceae | *Brosimum* | *utile* |
| Moraceae | *Ficus* | *americana* |
| Moraceae | *Ficus* | *broadwayi* |
| Moraceae | *Ficus* | *gomelleira* |
| Moraceae | *Ficus* | *malacocarpa* |
| Moraceae | *Ficus* | *maroniensis* |
| Moraceae | *Ficus* | *nymphaeifolia* |
| Moraceae | *Ficus* | *panurensis* |
| Moraceae | *Ficus* | *pertusa* |
| Moraceae | *Ficus* | *piresiana* |
| Moraceae | *Ficus* | *pulchella* |
| Moraceae | *Ficus* | *schumacheri* |
| Moraceae | *Helicostylis* | *pedunculata* |
| Moraceae | *Helicostylis* | *tomentosa* |
| Moraceae | *Maquira* | *guianensis* |
| Moraceae | *Naucleopsis* | *glabra* |
| Moraceae | *Naucleopsis* | *guianensis* |
| Moraceae | *Perebea* | *mollis* |
| Moraceae | *Perebea* | *rubra* |
| Moraceae | *Pseudolmedia* | *laevis* |
| Moraceae | *Trymatococcus* | *amazonicus* |
| Moraceae | *Trymatococcus* | *oligandrus* |
| Myristicaceae | *Iryanthera* | *hostmannii* |
| Myristicaceae | *Iryanthera* | *sagotiana* |
| Myristicaceae | *Virola* | *michelii* |
| Myristicaceae | *Virola* | *sebifera* |
| Myristicaceae | *Virola* | *surinamensis* |
| Myrtaceae | *Calycolpus* | *goetheanus* |
| Myrtaceae | *Eugenia* | *albicans* |
| Myrtaceae | *Eugenia* | *anastomosans* |
| Myrtaceae | *Eugenia* | *coffeifolia* |
| Myrtaceae | *Eugenia* | *cucullata* |
| Myrtaceae | *Eugenia* | *cupulata* |
| Myrtaceae | *Eugenia* | *exaltata* |
| Myrtaceae | *Eugenia* | *latifolia* |
| Myrtaceae | *Eugenia* | *patens* |
| Myrtaceae | *Eugenia* | *patrisii* |
| Myrtaceae | *Eugenia* | *pseudopsidium* |
| Myrtaceae | *Eugenia* | *sp.FG21-Holst* |
| Myrtaceae | *Eugenia* | *sp.FG9-Holst* |
| Myrtaceae | *Eugenia* | *tetramera* |
| Myrtaceae | *Myrcia* | *decorticans* |
| Myrtaceae | *Myrcia* | *fallax* |
| Myrtaceae | *Myrcia* | *magnoliifolia* |
| Myrtaceae | *Myrciaria* | *floribunda* |
| Myrtaceae | *Indet.* | *sp.B1* |
| Nyctaginaceae | *Neea* | *sp.1CAY-ATDN* |
| Nyctaginaceae | *Indet.* | *sp.4CAY-ATDN* |
| Nyctaginaceae | *Indet.* | *sp.7CAY-ATDN* |
| Nyctaginaceae | *Indet.* | *sp.P1* |
| Ochnaceae | *Elvasia* | *elvasioides* |
| Ochnaceae | *Lacunaria* | *crenata* |
| Ochnaceae | *Lacunaria* | *jenmanii* |
| Ochnaceae | *Ouratea* | *decagyna* |
| Ochnaceae | *Ouratea* | *guianensis* |
| Ochnaceae | *Quiina* | *guianensis* |
| Ochnaceae | *Quiina* | *integrifolia* |
| Ochnaceae | *Quiina* | *macrophylla* |
| Ochnaceae | *Quiina* | *obovata* |
| Ochnaceae | *Quiina* | *oiapocensis* |
| Ochnaceae | *Touroulia* | *guianensis* |
| Olacaceae | *Chaunochiton* | *kappleri* |
| Olacaceae | *Heisteria* | *densifrons* |
| Olacaceae | *Heisteria* | *ovata* |
| Olacaceae | *Minquartia* | *guianensis* |
| Opiliaceae | *Agonandra* | *silvatica* |
| Phyllanthaceae | *Amanoa* | *congesta* |
| Phyllanthaceae | *Amanoa* | *guianensis* |
| Phyllanthaceae | *Hieronyma* | *alchorneoides* |
| Phyllanthaceae | *Hieronyma* | *oblonga* |
| Phyllanthaceae | *Richeria* | *grandis* |
| Polygonaceae | *Coccoloba* | *mollis* |
| Primulaceae | *Cybianthus* | *guyanensis* |
| Primulaceae | *Cybianthus* | *microbotrys* |
| Proteaceae | *Euplassa* | *pinnata* |
| Proteaceae | *Panopsis* | *sessilifolia* |
| Putranjivaceae | *Drypetes* | *fanshawei* |
| Putranjivaceae | *Drypetes* | *variabilis* |
| Rhizophoraceae | *Cassipourea* | *guianensis* |
| Rosaceae | *Prunus* | *accumulans* |
| Rosaceae | *Prunus* | *myrtifolia* |
| Rubiaceae | *Amaioua* | *corymbosa* |
| Rubiaceae | *Amaioua* | *guianensis* |
| Rubiaceae | *Chimarrhis* | *turbinata* |
| Rubiaceae | *Coussarea* | *granvillei* |
| Rubiaceae | *Coussarea* | *machadoana* |
| Rubiaceae | *Coussarea* | *racemosa* |
| Rubiaceae | *Duroia* | *aquatica* |
| Rubiaceae | *Duroia* | *eriopila* |
| Rubiaceae | *Duroia* | *genipoides* |
| Rubiaceae | *Duroia* | *longiflora* |
| Rubiaceae | *Faramea* | *pedunculata* |
| Rubiaceae | *Faramea* | *sp.3* |
| Rubiaceae | *Ferdinandusa* | *paraensis* |
| Rubiaceae | *Isertia* | *coccinea* |
| Rubiaceae | *Kutchubaea* | *insignis* |
| Rubiaceae | *Palicourea* | *guianensis* |
| Rubiaceae | *Posoqueria* | *latifolia* |
| Rutaceae | *Zanthoxylum* | *acuminatum* |
| Rutaceae | *Zanthoxylum* | *ekmanii* |
| Salicaceae | *Casearia* | *combaymensis* |
| Salicaceae | *Casearia* | *decandra* |
| Salicaceae | *Casearia* | *guianensis* |
| Salicaceae | *Casearia* | *javitensis* |
| Salicaceae | *Casearia* | *pitumba* |
| Salicaceae | *Casearia* | *sp.1CAY-ATDN* |
| Salicaceae | *Casearia* | *sp.3CAY-ATDN* |
| Salicaceae | *Casearia* | *sp.5CAY-ATDN* |
| Salicaceae | *Casearia* | *sp.D* |
| Salicaceae | *Casearia* | *sylvestris* |
| Salicaceae | *Casearia* | *ulmifolia* |
| Salicaceae | *Hasseltia* | *floribunda* |
| Salicaceae | *Laetia* | *procera* |
| Sapindaceae | *Cupania* | *hirsuta* |
| Sapindaceae | *Cupania* | *rubiginosa* |
| Sapindaceae | *Cupania* | *scrobiculata* |
| Sapindaceae | *Matayba* | *arborescens* |
| Sapindaceae | *Matayba* | *inelegans* |
| Sapindaceae | *Matayba* | *opaca* |
| Sapindaceae | *Melicoccus* | *pedicellaris* |
| Sapindaceae | *Talisia* | *furfuracea* |
| Sapindaceae | *Talisia* | *hexaphylla* |
| Sapindaceae | *Talisia* | *megaphylla* |
| Sapindaceae | *Talisia* | *microphylla* |
| Sapindaceae | *Talisia* | *praealta* |
| Sapindaceae | *Talisia* | *simaboides* |
| Sapindaceae | *Talisia* | *sp.2CAY-ATDN* |
| Sapindaceae | *Toulicia* | *guianensis* |
| Sapindaceae | *Vouarana* | *guianensis* |
| Sapotaceae | *Chromolucuma* | *congestifolia* |
| Sapotaceae | *Chrysophyllum* | *argenteum* |
| Sapotaceae | *Chrysophyllum* | *cuneifolium* |
| Sapotaceae | *Chrysophyllum* | *pomiferum* |
| Sapotaceae | *Chrysophyllum* | *prieurii* |
| Sapotaceae | *Chrysophyllum* | *sanguinolentum* |
| Sapotaceae | *Chrysophyllum* | *sp.3CAY-ATDN* |
| Sapotaceae | *Chrysophyllum* | *sp.4* |
| Sapotaceae | *Chrysophyllum* | *venezuelanense* |
| Sapotaceae | *Ecclinusa* | *guianensis* |
| Sapotaceae | *Ecclinusa* | *ramiflora* |
| Sapotaceae | *Elaeoluma* | *nuda* |
| Sapotaceae | *Manilkara* | *bidentata* |
| Sapotaceae | *Manilkara* | *huberi* |
| Sapotaceae | *Micropholis* | *egensis* |
| Sapotaceae | *Micropholis* | *guyanensis* |
| Sapotaceae | *Micropholis* | *longipedicellata* |
| Sapotaceae | *Micropholis* | *melinoniana* |
| Sapotaceae | *Micropholis* | *mensalis* |
| Sapotaceae | *Micropholis* | *obscura* |
| Sapotaceae | *Micropholis* | *venulosa* |
| Sapotaceae | *Pouteria* | *ambelaniifolia* |
| Sapotaceae | *Pouteria* | *aubrevillei* |
| Sapotaceae | *Pouteria* | *bangii* |
| Sapotaceae | *Pouteria* | *bilocularis* |
| Sapotaceae | *Pouteria* | *caimito* |
| Sapotaceae | *Pouteria* | *cayennensis* |
| Sapotaceae | *Pouteria* | *cicatricata* |
| Sapotaceae | *Pouteria* | *coriacea* |
| Sapotaceae | *Pouteria* | *engleri* |
| Sapotaceae | *Pouteria* | *eugeniifolia* |
| Sapotaceae | *Pouteria* | *fimbriata* |
| Sapotaceae | *Pouteria* | *flavilatex* |
| Sapotaceae | *Pouteria* | *gongrijpii* |
| Sapotaceae | *Pouteria* | *grandis* |
| Sapotaceae | *Pouteria* | *guianensis* |
| Sapotaceae | *Pouteria* | *hispida* |
| Sapotaceae | *Pouteria* | *jariensis* |
| Sapotaceae | *Pouteria* | *melanopoda* |
| Sapotaceae | *Pouteria* | *oblanceolata* |
| Sapotaceae | *Pouteria* | *reticulata* |
| Sapotaceae | *Pouteria* | *retinervis* |
| Sapotaceae | *Pouteria* | *sagotiana* |
| Sapotaceae | *Pouteria* | *singularis* |
| Sapotaceae | *Pouteria* | *sp.19* |
| Sapotaceae | *Pouteria* | *sp.42CAY-ATDN* |
| Sapotaceae | *Pouteria* | *sp.46Guyafor* |
| Sapotaceae | *Pouteria* | *torta* |
| Sapotaceae | *Pouteria* | *venosa* |
| Sapotaceae | *Pradosia* | *cochlearia* |
| Sapotaceae | *Pradosia* | *ptychandra* |
| Sapotaceae | *Sarcaulus* | *brasiliensis* |
| Simaroubaceae | *Simaba* | *cedron* |
| Simaroubaceae | *Simaba* | *morettii* |
| Simaroubaceae | *Simaba* | *polyphylla* |
| Simaroubaceae | *Simarouba* | *amara* |
| Siparunaceae | *Siparuna* | *cuspidata* |
| Siparunaceae | *Siparuna* | *decipiens* |
| Stemonuraceae | *Discophora* | *guianensis* |
| Ulmaceae | *Ampelocera* | *edentula* |
| Urticaceae | *Cecropia* | *obtusa* |
| Urticaceae | *Cecropia* | *sciadophylla* |
| Urticaceae | *Coussapoa* | *angustifolia* |
| Urticaceae | *Coussapoa* | *asperifolia* |
| Urticaceae | *Pourouma* | *bicolor* |
| Urticaceae | *Pourouma* | *guianensis* |
| Urticaceae | *Pourouma* | *melinonii* |
| Urticaceae | *Pourouma* | *minor* |
| Urticaceae | *Pourouma* | *mollis* |
| Urticaceae | *Pourouma* | *villosa* |
| Violaceae | *Alsodeia* | *longiflora* |
| Violaceae | *Amphirrhox* | *longifolia* |
| Violaceae | *Leonia* | *glycycarpa* |
| Violaceae | *Paypayrola* | *guianensis* |
| Violaceae | *Rinorea* | *bahiensis* |
| Violaceae | *Rinorea* | *flavescens* |
| Violaceae | *Rinorea* | *guianensis* |
| Violaceae | *Rinorea* | *pectinosquamata* |
| Violaceae | *Rinorea* | *sp.1CAY-ATDN* |
| Vochysiaceae | *Qualea* | *dinizii* |
| Vochysiaceae | *Qualea* | *rosea* |
| Vochysiaceae | *Qualea* | *sp.1CAY-ATDN* |
| Vochysiaceae | *Ruizterania* | *albiflora* |
| Vochysiaceae | *Vochysia* | *guianensis* |
| Vochysiaceae | *Vochysia* | *surinamensis* |
| Vochysiaceae | *Vochysia* | *tomentosa* |
