# Supplementary material for: 30 Years of postdisturbance recruitment in a Neotropical forest
Source: Ecol Evol. 2021 Oct 7;11(21):14448–58. doi: 10.1002/ece3.7634 (PMC8571577; doi:10.1002/ece3.7634)
Supplement: Supplementary file 4 — Appendix S4 [file ECE3-11-14448-s004.docx]

30 YEARS OF POST-DISTURBANCE RECRUITMENT IN A NEOTROPICAL FOREST

APPENDIX 4

Mirabel A.^1^, Marcon E.^1^, Hérault B.^2, 3, 4^

1 UMR EcoFoG, AgroParistech, CNRS, Cirad, INRA, Université des Antilles, Université de Guyane.

2 CIRAD, UPR Forêts et Sociétés, Yamoussoukro, Côte d’Ivoire.

3 Forêts et Sociétés, Univ Montpellier, CIRAD, Montpellier, France

4 Institut National Polytechnique Félix Houphouët-Boigny, INP-HB, Yamoussoukro, Côte d’Ivoire.

Correspondence:

*Ariane Mirabel*

*Email:* [Ariane.Mirabel@gm](mailto:Ariane.Mirabel@ecofog.gf)ail.com

Appendix S4: Table of mean species trait values per species. NA values correspond to the case where no value is available for the species.

| **Species** | **Leaf thickness** | | **Leaf chlorophyll content** | | **Leaf toughness** | **SLA** | | | **WD** | | **Hmax** |
| --- | --- | --- | --- | --- | --- | --- | --- | --- | --- | --- | --- |
| *Abarema jupunba* | 222.6 | 69.38 | | 1.47 | | | 9 | 0.57 | | 36 | |
| *Abarema mataybifolia* | 300.68 | 63.85 | | 1.7 | | | 70.55 | 0.71 | | 26 | |
| *Agonandra silvatica* | 249.87 | 51.4 | | 1.57 | | | 161.7 | 0.67 | | 40 | |
| *Alexa wachenheimii* | 214.97 | 49.63 | | 1.37 | | | 215.5 | 0.51 | | 42 | |
| *Amaioua guianensis* | 191.47 | 50.13 | | 1.86 | | | 232.8 | 0.75 | | 11 | |
| *Ambelania acida* | 312.9 | 63.25 | | 2.36 | | | 279.35 | 0.42 | | 20 | |
| *Amphirrhox longifolia* | 168.57 | 43.6 | | 1.54 | | | 85.9 | 0.58 | | 25 | |
| *Anacardium spruceanum* | 150.87 | 40.83 | | 1.81 | | | 394.9 | 0.46 | | 40 | |
| *Antonia ovata* | 365.9 | 64.27 | | 2.22 | | | 143.8 | 0.41 | | 38 | |
| *Apeiba glabra* | 229.38 | 52.57 | | 1.02 | | | 151.6 | 0.46 | | 23 | |
| *Apeiba petoumo* | 228.05 | 46.82 | | 1.12 | | | 266.1 | 0.39 | | 30 | |
| *Aspidosperma album* | 341.52 | 62.15 | | 2.22 | | | 158.9 | 0.65 | | 35 | |
| *Aspidosperma cruentum* | 285.1 | 64.9 | | 2.18 | | | 202.25 | 0.74 | | 25 | |
| *Aspidosperma marcgravianum* | 226.67 | 54.87 | | 0.8 | | | 84.5 | 0.74 | | 45 | |
| *Astrocaryum sciophilum* | 430 | 74.15 | | 12.17 | | | 528 | NA | | 12 | |
| *Bagassa guianensis* | 314.1 | 39.03 | | 1.14 | | | 242.4 | 0.62 | | 45 | |
| *Balizia pedicellaris* | 187.3 | NA | | NA | | | 2.43 | 0.57 | | 44 | |
| *Bocoa prouacensis* | 218.87 | 51.18 | | 2.4 | | | 146.66 | 0.85 | | 34 | |
| *Bombacopsis nervosa* | 213.33 | 48.93 | | 1.15 | | | 162.8 | 0.52 | | 36 | |
| *Brosimum guianense* | 256.23 | 54.6 | | 1.74 | | | 40.5 | 0.72 | | 45 | |
| *Brosimum rubescens* | 211.28 | 57.03 | | 1.38 | | | 87.4 | 0.62 | | 32 | |
| *Brosimum utile* | 265.57 | 48.3 | | 2.11 | | | 241.9 | 0.62 | | 50 | |
| *Buchenavia grandis* | 163.67 | 52.23 | | 1.34 | | | 138 | 0.75 | | 30 | |
| *Byrsonima laevigata* | 330.5 | 52.17 | | 0.86 | | | 131.75 | 0.68 | | 23 | |
| *Capparis maroniensis* | 253.77 | 51.2 | | 1.75 | | | 259.49 | 0.61 | | 29 | |
| *Carapa procera* | 242.13 | 65.5 | | 2.59 | | | 578.15 | 0.55 | | 41 | |
| *Caryocar glabrum* | 171.9 | 48.17 | | 0.77 | | | 131.9 | 0.63 | | 48 | |
| *Casearia javitensis* | 147.65 | 50 | | 1.3 | | | 256.3 | 0.72 | | 17 | |
| *Casearia sylvestris* | 146.62 | 57.3 | | 1.38 | | | 68.05 | 0.72 | | 22 | |
| *Cassia spruceana* | 236.2 | 71.57 | | 1.52 | | | 79.2 | 0.76 | | 32 | |
| *Cassipourea guianensis* | 225.78 | 67.42 | | 0.91 | | | 110.3 | 0.69 | | 25 | |
| *Catostemma fragrans* | 271.33 | 56.3 | | 2.94 | | | 285.2 | 0.62 | | 25 | |
| *Cecropia obtusa* | 345.35 | 51.33 | | 1.71 | | | 1391.25 | 0.4 | | 24 | |
| *Chaetocarpus schomburgkianus* | 367.82 | 68.85 | | 2.85 | | | 185.1 | 0.78 | | 38 | |
| *Chaunochiton kappleri* | 276.1 | 47.07 | | 1.03 | | | 103.87 | 0.45 | | 32 | |
| *Cheiloclinium cognatum* | 157.05 | 50.85 | | 1.09 | | | 157.6 | 0.69 | | 10 | |
| *Chimarrhis turbinata* | 273.33 | 47.87 | | 1.31 | | | 318.6 | 0.61 | | 35 | |
| *Chrysophyllum argenteum* | 235.15 | 61.12 | | 0.94 | | | 71.85 | 0.78 | | 20 | |
| *Chrysophyllum cuneifolium* | 175.12 | 43.75 | | 1.29 | | | 69.8 | 0.76 | | 15 | |
| *Chrysophyllum pomiferum* | NA | NA | | NA | | | NA | NA | | 37 | |
| *Chrysophyllum prieurii* | 228.1 | 67.87 | | 2.03 | | | 228.4 | 0.81 | | 38 | |
| *Chrysophyllum sanguinolentum* | 293.45 | 53.77 | | 2.6 | | | 446.75 | 0.6 | | 40 | |
| *Coccoloba mollis* | 336.67 | 47.87 | | 1.39 | | | 1312.5 | 0.65 | | 30 | |
| *Conceveiba guianensis* | 202.95 | 56.98 | | 1.79 | | | 279.85 | 0.54 | | 20 | |
| *Cordia sagotii* | 344.23 | 57.43 | | 2.43 | | | 317.35 | 0.47 | | 15 | |
| *Couepia bracteosa* | 232.77 | 47.3 | | 2.07 | | | 294.6 | 0.8 | | 20 | |
| *Couepia caryophylloides* | 186.83 | 47.35 | | 1.86 | | | 317.95 | 0.76 | | 39 | |
| *Couepia guianensis* | 246.33 | 48.97 | | 1.59 | | | 99.46 | 0.74 | | 37 | |
| *Couepia habrantha* | 338.68 | 52.02 | | 2.47 | | | 164.9 | 0.77 | | 30 | |
| *Couepia parillo* | 201.42 | 46.97 | | 1.62 | | | 131.65 | 0.82 | | 20 | |
| *Couma guianensis* | 235.22 | 55.42 | | 1.09 | | | 177.78 | 0.47 | | 35 | |
| *Couratari calycina* | 292 | 62.03 | | 2.27 | | | 218.5 | 0.55 | | 31 | |
| *Couratari gloriosa* | 249.87 | 67.07 | | 2.03 | | | 294.4 | 0.61 | | 30 | |
| *Couratari guianensis* | 309.12 | 52.42 | | 1.95 | | | 151.21 | 0.46 | | 42 | |
| *Couratari multiflora* | 191.3 | 54.77 | | 1.4 | | | 61.1 | 0.62 | | 38 | |
| *Couratari oblongifolia* | 234.05 | 35.22 | | 1.25 | | | 64 | 0.48 | | 40 | |
| *Cupania scrobiculata* | 166.3 | 47.65 | | 1.19 | | | 143.25 | 0.71 | | 27 | |
| *Dacryodes nitens* | 257 | 61.07 | | 2.45 | | | 252.3 | 0.56 | | 23 | |
| *Dendrobangia boliviana* | 265.22 | 59.63 | | 0.97 | | | 152.54 | 0.62 | | 35 | |
| *Dialium guianense* | 181.85 | 46.77 | | 1.18 | | | 47.9 | 0.67 | | 35 | |
| *Dicorynia guianensis* | 244.87 | 59.57 | | 1.72 | | | 135.6 | 0.67 | | 52 | |
| *Diospyros capreifolia* | 180.53 | 55.6 | | 1.29 | | | 57.3 | 0.69 | | 25 | |
| *Diospyros carbonaria* | 303.43 | 61.83 | | 1.57 | | | 107.9 | 0.64 | | 20 | |
| *Diospyros cayennensis* | NA | NA | | NA | | | NA | NA | | 32 | |
| *Diplotropis purpurea* | 232.73 | 58.87 | | 1.49 | | | 160.1 | 0.79 | | 45 | |
| *Dipteryx odorata* | 261.37 | 45.4 | | 2.32 | | | 191.75 | 0.88 | | 40 | |
| *Drypetes fanshawei* | 204.68 | 57.77 | | 1.47 | | | 173.95 | 0.67 | | 33 | |
| *Drypetes variabilis* | 300.62 | 64.27 | | 2.21 | | | 177.35 | 0.71 | | 25 | |
| *Duguetia calycina* | 234.33 | 46.23 | | 2.01 | | | 252.6 | 0.67 | | 10 | |
| *Duguetia surinamensis* | 172.17 | 48.38 | | 1.63 | | | 111.55 | 0.73 | | 30 | |
| *Duroia aquatica* | 262.15 | 53.98 | | 2.25 | | | 1006.3 | 0.63 | | 15 | |
| *Duroia eriopila* | 271.57 | 52.87 | | 1.97 | | | 437.42 | 0.7 | | 15 | |
| *Ecclinusa ramiflora* | 248.38 | 49.49 | | 2.03 | | | 200.9 | 0.6 | | 30 | |
| *Emmotum fagifolium* | 215.95 | 61.33 | | 1.46 | | | 108.1 | 0.7 | | 30 | |
| *Endlicheria melinonii* | 301.57 | 42.17 | | 1.82 | | | 173.6 | 0.6 | | 25 | |
| *Enterolobium schomburgkii* | NA | NA | | NA | | | 2.1 | 0.71 | | 40 | |
| *Eperua falcata* | 163.83 | 51.03 | | 1.22 | | | 99.8 | 0.65 | | 44 | |
| *Eperua grandiflora* | 282.87 | 55.83 | | 2.49 | | | 92.9 | 0.67 | | 42 | |
| *Eschweilera apiculata* | 213.33 | 56.53 | | 1.71 | | | 115.01 | 0.67 | | 38 | |
| *Eschweilera chartaceifolia* | 176 | 54.7 | | 1.38 | | | 168.05 | 0.7 | | 28 | |
| *Eschweilera congestiflora* | 270.83 | 71 | | 2.41 | | | 495.8 | 0.73 | | 32 | |
| *Eschweilera coriacea* | 209.23 | 58.7 | | 1.89 | | | 231.05 | 0.69 | | 37 | |
| *Eschweilera decolorans* | 254.43 | 58.5 | | 2.31 | | | 180.55 | 0.75 | | 38 | |
| *Eschweilera grandiflora* | 245.7 | 56.3 | | 2.33 | | | 119.87 | 0.68 | | 42 | |
| *Eschweilera micrantha* | 182.9 | 51.43 | | 1.78 | | | 173.2 | 0.74 | | 37 | |
| *Eschweilera parviflora* | 204.08 | 50.35 | | 1.8 | | | 173 | 0.72 | | 43 | |
| *Eschweilera pedicellata* | 191.67 | 44.07 | | 1.58 | | | 146.3 | 0.69 | | 20 | |
| *Eschweilera sagotiana* | 310.83 | 57.28 | | 2.91 | | | 169.2 | 0.71 | | 41 | |
| *Eschweilera simiorum* | 172 | 57.13 | | 1.58 | | | 416.7 | 0.68 | | 25 | |
| *Eschweilera squamata* | 195.88 | 57.88 | | 1.44 | | | 180.7 | 0.61 | | 35 | |
| *Eugenia coffeifolia* | 175.33 | 63.5 | | 1.3 | | | 51.2 | 0.79 | | 25 | |
| *Eugenia macrocalyx* | 263.22 | 61.13 | | 1.45 | | | 101.01 | 0.78 | | 25 | |
| *Eugenia patrisii* | 157.57 | 50.23 | | 0.85 | | | 30.8 | 0.74 | | 15 | |
| *Eugenia pseudopsidium* | 209.1 | 60.95 | | 1.29 | | | 94.74 | 0.7 | | 25 | |
| *Eugenia tetramera* | 216.82 | 51.92 | | 1.26 | | | 296.6 | 0.76 | | 25 | |
| *Euterpe oleracea* | 224.3 | 56 | | 3.32 | | | 355.2 | NA | | 28 | |
| *Fusaea longifolia* | 194.23 | 53.4 | | 1.6 | | | 151.8 | 0.63 | | 12 | |
| *Geissospermum laeve* | 153.23 | 61.6 | | 1.33 | | | 82.2 | 0.72 | | 46 | |
| *Glycydendron amazonicum* | 161.37 | 56.23 | | 1.46 | | | 169.1 | 0.55 | | 36 | |
| *Goupia glabra* | 172.78 | 57.4 | | 1.4 | | | 81.2 | 0.66 | | 42 | |
| *Guatteria guianensis* | 244.52 | 52.17 | | 2.19 | | | 1380.8 | 0.58 | | 15 | |
| *Gustavia hexapetala* | 210 | 53.13 | | 1.42 | | | 186.4 | 0.63 | | 20 | |
| *Hebepetalum humiriifolium* | 212.47 | 57.63 | | 1.17 | | | 266.6 | 0.72 | | 20 | |
| *Heisteria densifrons* | 194.23 | 42.5 | | 1.88 | | | 178.1 | 0.84 | | 16 | |
| *Helicostylis pedunculata* | 258.83 | 50 | | 1.74 | | | 181.6 | 0.54 | | 25 | |
| *Helicostylis tomentosa* | 329.1 | 53 | | 2.31 | | | 189.3 | 0.6 | | 30 | |
| *Hevea guianensis* | 180.17 | 56.43 | | 1.75 | | | 130.8 | 0.57 | | 35 | |
| *Hirtella bicornis* | 164.37 | 49.25 | | 1.35 | | | 35.15 | 0.74 | | 29 | |
| *Hirtella glandulosa* | 285.23 | 50.83 | | 1.78 | | | 261.5 | 0.78 | | 35 | |
| *Humiriastrum subcrenatum* | 307.8 | 63.13 | | 1.79 | | | 23.2 | 0.7 | | 48 | |
| *Hymenolobium flavum* | 162.1 | 39.77 | | 0.88 | | | 13.5 | 0.66 | | 50 | |
| *Inga alba* | 158.28 | 59.78 | | 1.37 | | | 104.25 | 0.51 | | 40 | |
| *Inga fanchoniana* | 188.53 | 54.43 | | 1.43 | | | 471.5 | 0.78 | | 37 | |
| *Inga gracilifolia* | 230.67 | 71.87 | | 1 | | | 30.55 | 0.81 | | 35 | |
| *Inga huberi* | 216.67 | 64.3 | | 1.5 | | | 296.25 | 0.64 | | 21 | |
| *Inga leiocalycina* | 188.1 | 37.53 | | 1.1 | | | 191.7 | 0.7 | | 25 | |
| *Inga melinonis* | 276.83 | 61.43 | | 1.69 | | | 314.17 | 0.68 | | 29 | |
| *Inga paraensis* | 200.63 | 69.43 | | 1.68 | | | 150.3 | 0.63 | | 38 | |
| *Inga pezizifera* | 154.23 | 57.5 | | 1.41 | | | 200.9 | 0.55 | | 30 | |
| *Inga rubiginosa* | 258.2 | 65.8 | | 1.42 | | | 236.4 | 0.61 | | 21 | |
| *Inga sarmentosa* | 263.33 | 79.8 | | 2.45 | | | 274 | 0.64 | | 25 | |
| *Inga stipularis* | 299.47 | 66.23 | | 1.61 | | | 109.8 | 0.73 | | 23 | |
| *Iryanthera hostmannii* | 203.43 | 63.9 | | 0.94 | | | 187 | 0.57 | | 13 | |
| *Iryanthera sagotiana* | 230.38 | 60.73 | | 0.83 | | | 112.8 | 0.54 | | 31 | |
| *Jacaranda copaia* | 257.77 | 63.97 | | 0.77 | | | 87.5 | 0.38 | | 38 | |
| *Lacistema grandifolium* | 235.67 | 52.4 | | 1.45 | | | 147.09 | 0.49 | | 15 | |
| *Lacmellea aculeata* | 247.72 | 63.43 | | 0.99 | | | 153.75 | 0.54 | | 20 | |
| *Lacunaria crenata* | 158.57 | 52.77 | | 1.42 | | | 94.65 | 0.8 | | 23 | |
| *Laetia procera* | 223.32 | 67.82 | | 1.6 | | | 166.91 | 0.58 | | 30 | |
| *Lecythis chartacea* | 146.05 | 50.65 | | 1.21 | | | 145.27 | 0.69 | | 40 | |
| *Lecythis corrugata* | 255 | 52.93 | | 1.95 | | | 146.8 | 0.58 | | 35 | |
| *Lecythis holcogyne* | 246.67 | 55.18 | | 1.7 | | | 112.66 | 0.64 | | 40 | |
| *Lecythis idatimon* | 248.43 | 54.9 | | 2.03 | | | 133.4 | 0.63 | | 44 | |
| *Lecythis persistens* | 310 | 58.37 | | 2.42 | | | 203.3 | 0.7 | | 30 | |
| *Lecythis poiteaui* | 182.77 | 46.57 | | 1.35 | | | 141.25 | 0.66 | | 38 | |
| *Lecythis zabucajo* | 183.77 | 45.07 | | 0.9 | | | 71.6 | 0.69 | | 55 | |
| *Leonia glycycarpa* | 226.6 | 62.08 | | 1.95 | | | 185.5 | 0.54 | | 23 | |
| *Licania alba* | 324.62 | 50.97 | | 2.69 | | | 284.6 | 0.77 | | 37 | |
| *Licania canescens* | 195.87 | 54.3 | | 1.49 | | | 81.7 | 0.78 | | 33 | |
| *Licania heteromorpha* | 240.9 | 55.08 | | 1.86 | | | 93.81 | 0.78 | | 35 | |
| *Licania kunthiana* | 324.33 | 52.35 | | 1.83 | | | 69.36 | 0.8 | | 35 | |
| *Licania latistipula* | 287.13 | 55.47 | | 2 | | | 277.9 | 0.79 | | 39 | |
| *Licania laxiflora* | 260.35 | 48.52 | | 2.17 | | | 57.05 | 0.83 | | 29 | |
| *Licania licaniiflora* | 225.62 | 60.57 | | 2.26 | | | 316.65 | 0.69 | | 30 | |
| *Licania membranacea* | 260.78 | 50.02 | | 1.99 | | | 128.3 | 0.8 | | 40 | |
| *Licania micrantha* | 274.2 | 54.7 | | 2.42 | | | 133.6 | 0.8 | | 30 | |
| *Licania ovalifolia* | 429.45 | 66.12 | | 2.92 | | | 131.8 | 0.81 | | 37 | |
| *Licania sprucei* | 264.23 | 63.23 | | 3.32 | | | 93.07 | 0.67 | | 20 | |
| *Licaria cannella* | 240.83 | 55 | | 2.09 | | | 161.4 | 0.69 | | 40 | |
| *Licaria chrysophylla* | 252.38 | 52.68 | | 2.24 | | | 141.25 | 0.71 | | 36 | |
| *Licaria guianensis* | 161.57 | 48.17 | | 1.44 | | | 57.7 | 0.81 | | 25 | |
| *Loreya arborescens* | 200 | 61.7 | | 0.93 | | | 132.16 | 0.53 | | 37 | |
| *Lueheopsis rugosa* | 346.83 | 48.33 | | 1.94 | | | 437.5 | 0.52 | | 35 | |
| *Mabea piriri* | 189.22 | 69.47 | | 1.35 | | | 125.3 | 0.62 | | 20 | |
| *Macoubea guianensis* | 195.93 | 48.58 | | 0.85 | | | 198.9 | 0.41 | | 32 | |
| *Macrolobium bifolium* | 160 | 42.67 | | 1.54 | | | 275.3 | 0.71 | | 31 | |
| *Manilkara bidentata* | 325 | 65.17 | | 2.28 | | | 206.9 | 0.74 | | 50 | |
| *Manilkara huberi* | 327.67 | 60.4 | | 2.19 | | | 282.9 | 0.73 | | 50 | |
| *Maquira guianensis* | 201.2 | 68.4 | | 1.58 | | | 107.48 | 0.71 | | 30 | |
| *Maytenus oblongata* | 282.22 | 62 | | 2.07 | | | 244.85 | 0.74 | | 13 | |
| *Miconia acuminata* | 209.32 | 57.17 | | 0.63 | | | 154.55 | 0.66 | | 20 | |
| *Miconia fragilis* | 173.33 | 47.3 | | 0.61 | | | 138.72 | 0.58 | | 18 | |
| *Miconia tschudyoides* | 259.57 | 48.37 | | 0.78 | | | 382.8 | 0.53 | | 10 | |
| *Micropholis egensis* | 310.43 | 53.6 | | 1.39 | | | 110.1 | 0.62 | | 40 | |
| *Micropholis guyanensis* | 254.67 | 53.4 | | 1.43 | | | 119.2 | 0.64 | | 42 | |
| *Micropholis obscura* | 293.6 | 53.63 | | 1.81 | | | 79.35 | 0.7 | | 44 | |
| *Micropholis venulosa* | 161.95 | 50.72 | | 1.03 | | | 33.3 | 0.58 | | 40 | |
| *Minquartia guianensis* | 170.67 | 48.6 | | 1.74 | | | 141.16 | 0.68 | | 37 | |
| *Moronobea coccinea* | 254.7 | 64.1 | | 0.83 | | | 45.5 | 0.62 | | 40 | |
| *Mouriri crassifolia* | 310.67 | 66.1 | | 3.06 | | | 109.95 | 0.78 | | 35 | |
| *Mouriri huberi* | 299.13 | 52.5 | | 1.61 | | | 71.72 | 0.7 | | 30 | |
| *Myrcia decorticans* | 282.5 | 60.62 | | 1.75 | | | 97.35 | 0.75 | | 30 | |
| *Myrciaria floribunda* | 133.33 | 50.2 | | 0.72 | | | 168 | 0.71 | | 25 | |
| *Naucleopsis guianensis* | 314.77 | 55.17 | | 1.79 | | | 113.14 | 0.55 | | 20 | |
| *Neea floribunda* | 271 | 61.1 | | 1.04 | | | 233.5 | 0.4 | | 18 | |
| *Ocotea amazonica* | 237.47 | 55.73 | | 1.63 | | | 158.5 | 0.51 | | 25 | |
| *Ocotea argyrophylla* | 273.53 | 64.37 | | 1.4 | | | 127 | 0.43 | | NA | |
| *Ocotea indirectinervia* | 309.28 | 68.57 | | 3.44 | | | 162.8 | 0.59 | | 33 | |
| *Ocotea tomentella* | 268.13 | 53.43 | | 1.93 | | | 478.2 | 0.5 | | 46 | |
| *Oenocarpus bacaba* | 325 | 75.3 | | 5.82 | | | 650.4 | NA | | 25 | |
| *Ormosia coccinea* | 304.22 | 46.62 | | 2.6 | | | 275.13 | 0.48 | | 43 | |
| *Ouratea melinonii* | 144.3 | 50.73 | | 1.13 | | | 74.5 | 0.68 | | 24 | |
| *Oxandra asbeckii* | 221.67 | 61.78 | | 1.73 | | | 85.25 | 0.78 | | 18 | |
| *Pachira dolichocalyx* | 201.33 | 55.2 | | 1.23 | | | 100.2 | 0.53 | | 25 | |
| *Palicourea guianensis* | 193.32 | 67.18 | | 1.16 | | | 734.4 | 0.51 | | 12 | |
| *Parahancornia fasciculata* | 220.8 | 63.67 | | 0.77 | | | 65.2 | 0.45 | | 33 | |
| *Parinari campestris* | 324.8 | 42.88 | | 2.52 | | | 63.4 | 0.7 | | 25 | |
| *Parinari excelsa* | 225.28 | 56.98 | | 1.85 | | | 116.2 | 0.62 | | 40 | |
| *Parinari montana* | 341.1 | 47.8 | | 2.77 | | | 114.37 | 0.7 | | 39 | |
| *Parkia nitida* | 233.67 | 62.7 | | NA | | | 1.13 | 0.69 | | 40 | |
| *Parkia pendula* | NA | NA | | NA | | | NA | NA | | 39 | |
| *Parkia ulei* | 127.15 | 48.35 | | 0.41 | | | 0.03 | 0.62 | | 45 | |
| *Parkia velutina* | 161.53 | 52.13 | | NA | | | 0.49 | 0.47 | | 40 | |
| *Peltogyne paniculata* | 112.33 | 43.6 | | 0.96 | | | 56.7 | 0.86 | | 30 | |
| *Peltogyne venosa* | 243 | 70.37 | | 1.53 | | | 85.92 | NA | | 45 | |
| *Perebea rubra* | 263.43 | 50.87 | | 1.61 | | | 514.1 | 0.6 | | 30 | |
| *Platonia insignis* | 446.43 | 55.63 | | 2.56 | | | 183.8 | 0.59 | | 40 | |
| *Platymiscium pinnatum* | 180.45 | 50.22 | | 1.6 | | | 204.54 | 0.74 | | 35 | |
| *Pogonophora schomburgkiana* | 202.38 | 61.18 | | 1.58 | | | 114.3 | 0.79 | | 20 | |
| *Poraqueiba guianensis* | 223.02 | 54.08 | | 1.69 | | | 218.2 | 0.67 | | 29 | |
| *Posoqueria latifolia* | 344.67 | 64.33 | | 1.93 | | | 299.2 | 0.62 | | 15 | |
| *Pourouma bicolor* | 317.83 | 52.38 | | 1.91 | | | 203.45 | 0.35 | | 15 | |
| *Pourouma melinonii* | 301.3 | 57.13 | | 2.56 | | | 329.5 | 0.42 | | 28 | |
| *Pourouma villosa* | 343.78 | 51.53 | | 2.42 | | | 305.75 | 0.42 | | 25 | |
| *Pouteria ambelaniifolia* | 344.8 | 61.2 | | 3.14 | | | 217.6 | 0.81 | | 30 | |
| *Pouteria bilocularis* | 235 | 63.62 | | 1.61 | | | 72.45 | 0.75 | | 32 | |
| *Pouteria egregia* | 266.8 | 62.58 | | 1.15 | | | 40.7 | 0.73 | | 43 | |
| *Pouteria engleri* | 190.9 | 59.48 | | 1.36 | | | 105.11 | 0.63 | | 35 | |
| *Pouteria eugeniifolia* | 249.1 | 54.23 | | 1.53 | | | 38.35 | 0.72 | | 41 | |
| *Pouteria fimbriata* | 132.67 | 57.17 | | 1.3 | | | 266.75 | 0.8 | | 35 | |
| *Pouteria gonggrijpii* | 262.98 | 53.9 | | 2.23 | | | 213 | 0.7 | | 28 | |
| *Pouteria grandis* | 338.67 | 67.65 | | 2.33 | | | 58.56 | 0.72 | | 44 | |
| *Pouteria guianensis* | 275.43 | 60.7 | | 2.05 | | | 134 | 0.78 | | 35 | |
| *Pouteria hispida* | 203.33 | 52.43 | | 1.89 | | | 149.05 | 0.77 | | 36 | |
| *Pouteria jariensis* | 242.33 | 52.95 | | 2.1 | | | 105.28 | 0.7 | | 26 | |
| *Pouteria reticulata* | 252 | 45.07 | | 2.05 | | | 103.8 | 0.67 | | 37 | |
| *Pouteria singularis* | 179.12 | 52.48 | | 1.28 | | | 134.65 | 0.71 | | 20 | |
| *Pouteria torta* | 234.57 | 52.2 | | 2.06 | | | 152.66 | 0.79 | | 35 | |
| *Pouteria venosa* | 118.43 | 53.03 | | 0.65 | | | 186.5 | NA | | 40 | |
| *Pradosia cochlearia* | 274.67 | 49.37 | | 1.76 | | | 40.9 | 0.68 | | 49 | |
| *Pradosia ptychandra* | 180.9 | 54.27 | | 1.39 | | | 133.3 | 0.62 | | 37 | |
| *Protium apiculatum* | 173.45 | 55.35 | | 2.03 | | | 277.55 | 0.55 | | 25 | |
| *Protium decandrum* | 218.23 | 63.47 | | 2.07 | | | 107.75 | 0.54 | | 41 | |
| *Protium gallicum* | 165.97 | 47.57 | | 1.09 | | | 50.45 | 0.63 | | 35 | |
| *Protium giganteum* | 196.67 | 64.5 | | 2.55 | | | 137.95 | 0.44 | | 30 | |
| *Protium guianense* | 168.95 | 51.27 | | 1.2 | | | 46.05 | 0.7 | | 45 | |
| *Protium opacum* | 198.3 | 50.87 | | 2.14 | | | 168.8 | 0.57 | | 30 | |
| *Protium sagotianum* | 184.88 | 56.27 | | 2.04 | | | 261.6 | 0.62 | | 23 | |
| *Protium subserratum* | 186.7 | 53.23 | | 1.31 | | | 139.1 | 0.46 | | 33 | |
| *Protium tenuifolium* | 248.77 | 56.67 | | 3 | | | 290.7 | 0.6 | | 34 | |
| *Protium trifoliolatum* | 158.93 | 49.53 | | 1.34 | | | 85.8 | 0.64 | | 15 | |
| *Pseudopiptadenia suaveolens* | 209.57 | 42 | | NA | | | 0.4 | 0.65 | | 41 | |
| *Qualea rosea* | 273.33 | 65.83 | | 1.39 | | | 102.7 | 0.61 | | 46 | |
| *Quiina guianensis* | 128.33 | 44.53 | | 1.32 | | | 238.6 | 0.84 | | 10 | |
| *Quiina obovata* | 214.88 | 56 | | 2.05 | | | 501.01 | 0.77 | | 15 | |
| *Recordoxylon speciosum* | 172.05 | 52.28 | | 1.19 | | | 126.9 | 0.92 | | 40 | |
| *Rhabdodendron amazonicum* | 367.33 | 47.6 | | 1.7 | | | 107.26 | 0.56 | | 10 | |
| *Rhodostemonodaphne grandis* | 275.57 | 63.47 | | 3.27 | | | 365.7 | 0.43 | | 30 | |
| *Ruizterania albiflora* | 195.73 | 55.12 | | 0.94 | | | 45.75 | 0.63 | | 53 | |
| *Sacoglottis cydonioides* | 258.87 | 38.77 | | 1.93 | | | 140.7 | 0.68 | | 35 | |
| *Sacoglottis guianensis* | 315.52 | 56.4 | | 2.25 | | | 93.85 | 0.71 | | 37 | |
| *Sandwithia guianensis* | 195.37 | 55.27 | | 1.4 | | | 156.21 | 0.7 | | 14 | |
| *Schefflera decaphylla* | 335.62 | 65.08 | | 2.04 | | | 210.65 | 0.45 | | 40 | |
| *Schefflera morototoni* | 284.23 | 57.77 | | 0.99 | | | 104.4 | 0.51 | | 35 | |
| *Sextonia rubra* | 250.67 | 62.08 | | 1.58 | | | 179.25 | 0.46 | | 44 | |
| *Simaba cedron* | 338.72 | 71.8 | | 4.01 | | | 253 | 0.42 | | 10 | |
| *Simaba morettii* | 283.33 | 55.8 | | 1.71 | | | 28 | 0.38 | | 40 | |
| *Simarouba amara* | 394.2 | 62.8 | | 1.1 | | | 86 | 0.33 | | 41 | |
| *Siparuna decipiens* | 284.1 | 56.3 | | 1.23 | | | 130.3 | 0.57 | | 18 | |
| *Sloanea guianensis* | 198.45 | 49.02 | | 1.71 | | | 222.47 | 0.69 | | 40 | |
| *Sterculia pruriens* | 252 | 50.52 | | 1.87 | | | 310.6 | 0.45 | | 43 | |
| *Sterculia speciosa* | 379.22 | 47.1 | | 3.04 | | | 692.65 | 0.5 | | 43 | |
| *Stryphnodendron polystachyum* | 182.57 | 51.63 | | 1.38 | | | 73.1 | 0.58 | | 40 | |
| *Swartzia arborescens* | 127.77 | 52.58 | | 0.87 | | | 48.95 | 0.71 | | 23 | |
| *Swartzia grandifolia* | 283.67 | 53.07 | | 2.93 | | | 135.9 | 0.66 | | 18 | |
| *Swartzia leblondii* | 228.97 | 53.92 | | 1.83 | | | 161.65 | 0.84 | | 23 | |
| *Swartzia oblanceolata* | 212 | 50.62 | | 1.71 | | | 113.54 | 0.64 | | 30 | |
| *Swartzia panacoco* | 240.4 | 37.2 | | 1.83 | | | 196.85 | 0.83 | | 33 | |
| *Swartzia polyphylla* | 242.1 | 59.5 | | 1.41 | | | 74.2 | 0.68 | | 44 | |
| *Symphonia globulifera* | 304.47 | 64.07 | | 1.31 | | | 83.1 | 0.56 | | 39 | |
| *Tabebuia capitata* | 296.67 | 31.87 | | 0.65 | | | 138.29 | 0.77 | | 32 | |
| *Tabebuia serratifolia* | 113.33 | 38.33 | | 0.86 | | | 219.13 | 0.65 | | 30 | |
| *Tachigali guianensis* | 152.23 | 31.67 | | 0.75 | | | 105.9 | 0.42 | | 40 | |
| *Tachigali melinonii* | 151.67 | 52.42 | | 1.28 | | | 102.34 | 0.73 | | 51 | |
| *Talisia hexaphylla* | 254.07 | 56.25 | | 2.09 | | | 90.28 | 0.76 | | 30 | |
| *Talisia microphylla* | 195 | 54.57 | | 1.04 | | | 62.35 | 0.8 | | 25 | |
| *Talisia praealta* | 212.77 | 61.47 | | 1.69 | | | 93.66 | 0.74 | | 30 | |
| *Tapirira bethanniana* | 213 | 62 | | 1.09 | | | 162.22 | 0.58 | | 50 | |
| *Tapirira guianensis* | 181.6 | 64.18 | | 1.23 | | | 116.28 | 0.56 | | 25 | |
| *Tapirira obtusa* | 187.13 | 55.17 | | 1.02 | | | 146.9 | 0.46 | | 35 | |
| *Tapura amazonica* | 250.13 | 70.33 | | 2.41 | | | 230.1 | 0.63 | | 8 | |
| *Tapura capitulifera* | 388 | 64.45 | | 3.23 | | | 55.85 | 0.72 | | 35 | |
| *Tapura guianensis* | 313.45 | 54.33 | | 1.83 | | | 128.41 | 0.59 | | 5 | |
| *Tetragastris panamensis* | 206.97 | 59.33 | | 1.85 | | | 96.65 | 0.69 | | 39 | |
| *Theobroma subincanum* | 305.33 | 54.72 | | 2.1 | | | 181.3 | 0.56 | | 20 | |
| *Theobroma velutinum* | 340 | 54.8 | | 3.67 | | | 83.66 | 0.62 | | 15 | |
| *Thyrsodium guianense* | 248.33 | 56.93 | | 1.99 | | | 88.88 | 0.61 | | 35 | |
| *Thyrsodium puberulum* | 238.1 | 51.98 | | 1.95 | | | 284.25 | 0.52 | | 34 | |
| *Touroulia guianensis* | 209.33 | 52.87 | | 1.86 | | | 40 | 0.72 | | 30 | |
| *Trichilia micrantha* | 161.17 | 55.15 | | 0.75 | | | 148.6 | 0.63 | | 20 | |
| *Trichilia schomburgkii* | 268 | 60 | | 1.66 | | | 238.6 | 0.78 | | 20 | |
| *Trymatococcus amazonicus* | 227.33 | 63.93 | | 1.46 | | | 205.5 | 0.55 | | 30 | |
| *Trymatococcus oligandrus* | 224.47 | 60.38 | | 1.93 | | | 50 | 0.55 | | 20 | |
| *Unonopsis rufescens* | 194.62 | 60.33 | | 1.72 | | | 170.8 | 0.58 | | 18 | |
| *Vantanea parviflora* | 273.72 | 64.63 | | 1.56 | | | 76.61 | 0.79 | | 40 | |
| *Vatairea erythrocarpa* | 233.27 | 51.28 | | 2 | | | 70.65 | 0.72 | | 30 | |
| *Virola michelii* | 202.9 | 62.82 | | 0.9 | | | 114.35 | 0.46 | | 41 | |
| *Vismia cayennensis* | 205.8 | 52.9 | | 1.41 | | | 96.6 | 0.54 | | 10 | |
| *Vochysia guianensis* | 233 | 56.03 | | 1.49 | | | 128.7 | 0.49 | | 42 | |
| *Vochysia tomentosa* | 286.67 | 56.12 | | 1.46 | | | 132.22 | 0.34 | | 32 | |
| *Vouacapoua americana* | 148.45 | 47.67 | | 1.44 | | | 119.1 | 0.68 | | 38 | |
| *Xylopia nitida* | 260 | 63.87 | | 1.56 | | | 78.7 | 0.56 | | 34 | |
| *Zygia racemosa* | 242.77 | 67.38 | | 1.5 | | | 2.73 | 0.74 | | 30 | |
| *Zygia tetragona* | 119.9 | NA | | NA | | | 0.48 | 0.88 | | 35 | |
